# Diverse Filters to Sense: Great Variability of Antennal Morphology and Sensillar Equipment in Gall-Wasps (Hymenoptera: Cynipidae)

**DOI:** 10.1371/journal.pone.0101843

**Published:** 2014-07-08

**Authors:** Carlo Polidori, José L. Nieves-Aldrey

**Affiliations:** Departamento de Biodiversidad y Biología Evolutiva, Museo Nacional de Ciencias Naturales (CSIC), Madrid, Spain; University of Arizona, United States of America

## Abstract

Comparative studies on antennal sensillar equipment in insects are largely lacking, despite their potential to provide insights into both ecological and phylogenetic relationships. Here we present the first comparative study on antennal morphology and sensillar equipment in female Cynipoidea (Hymenoptera), a large and diverse group of wasps, with special reference to the so-called gall-wasps (Cynipidae). A SEM analysis was conducted on 51 species from all extant cynipoid families and all cynipid tribes, and spanning all known life-histories in the superfamily (gall-inducers, gall-inquilines, and non-gall associated parasitoids). The generally filiform, rarely clavate, antennal flagellum of Cynipoidea harbours overall 12 types of sensilla: s. placoidea (SP), two types of s. coeloconica (SCo-A, SCo-B), s. campaniformia (SCa), s. basiconica (SB), five types of s. trichoidea (ST-A, B, C, D, E), large disc sensilla (LDS) and large volcano sensilla (LVS). We found a great variability in sensillar equipment both among and within lineages. However, few traits seem to be unique to specific cynipid tribes. Paraulacini are, for example, distinctive in having apical LVS; Pediaspidini are unique in having ≥3 rows of SP, each including 6–8 sensilla per flagellomere, and up to 7 SCo-A in a single flagellomere; Eschatocerini have by far the largest SCo-A. Overall, our data preliminarily suggest a tendency to decreased numbers of SP rows per flagellomere and increased relative size of SCo-A during cynipoid evolution. Furthermore, SCo-A size seems to be higher in species inducing galls in trees than in those inducing galls in herbs. On the other hand, ST seem to be more abundant on the antennae of herb-gallers than wood-gallers. The antennal morphology and sensillar equipment in Cynipoidea are the complex results of different interacting pressures that need further investigations to be clarified.

## Introduction

The antennae play a crucial role in the life of insects [1], since they receive stimuli related to food location, nest location, inter- and intra-specific recognition, mating and suitability of environmental conditions [2]–[4]. Specialized receptors that form part of the antennal epidermis, the antennal sensilla, comprise the link between stimuli and behavior. Sensilla consist of cuticular components, sensory neuron(s) and sheath cells, and greatly vary in shape, including long and short hair-like or plate-like structures, which may have single or double cuticular walls and which may be aporous, single-porous or multi-porous [5]. The different sensilla types also vary in their function, which can be mechanical, olfactory, gustatory, CO_2_-sensing or hygrothermal, thus playing different roles in perceiving physical, chemical and/or chemotactile stimuli [6]. Insect sensilla have been identified and classified within the limits of resolution of light and scanning electron microscopy by external morphology and through histological studies and transmission electron microscopy by internal structure [2], [6]–[8].

Apart from variation in morphology among sensilla types, there is also important variability in the incidence, density, and distribution of the different types of sensilla among species, even within a single genus, and between sexes within a species [9]–[11]. Besides possible – though still not much explored – phylogenetic effects on such variability [12], the diversity, density, and distribution patterns of sensilla may be the product of interacting selection pressures related to feeding and foraging habits, habitat type, mating systems, and social behaviour [13]–[15].

Within Hymenoptera, studies on antennal sensilla are numerous and have a long tradition, with possibly the oldest dating back to the 50's of the 19^th^ century [16]. Detailed information on the external, and more rarely internal, morphology and diversity of sensillar equipment on hymenopteran antennae is available in particular for parasitoid wasps in the superfamilies Ichneumonoidea and Chalcidoidea [11], [17]–[29]. In addition, some studies have analysed antennal sensilla in pollinivorous and predaceous Hymenoptera, particularly Apoidea (Aculeata) (and most often bees) [9], [30]–[33].

Most of studies on hymenopteran sensilla, however, deal with only one or a few species, making it difficult to compare similar equipment within taxonomic groups, particularly because the terminology is inconsistent across published papers. In fact, morphological comparisons involving many species within specific lineages (beyond the order-level) have apparently been attempted only for Formicidae (ants) [34], for *Bombus* species of bees [35], for Philanthinae digger wasps [33], for Emphorini bees [32] and for fig wasps (Chalcidoidea: Agaonidae) [36], i.e. mostly for Aculeata (the exceptions are fig wasps). A few additional studies concern comparisons of the density of certain types of sensilla and/or of the antennal morphology, rather than a complete overview of sensilla morphology and diversity [15], [37], [38]. Other groups have largely been ignored to date in such studies. Here, we present the fist large, comparative study on female Cynipoidea, in order to provide new data on the diversity of antennal morphology and sensillar equipment in the Hymenoptera as a whole.

The Cynipoidea is a large family of Hymenoptera (>3000 species) in the infra-order Proctotrupomorpha [39], [40]. The superfamily includes basal parasitoid lineages (Ibaliidae, Liopteridae) and two derived, extremely rich and diverse families: the parasitoid Figitidae and the secondarily herbivorous Cynipidae. The latter family includes the so-called gall-wasps, characterized by a life-cycle that includes the formation (or in some cases the usurpation (inquilinism)) of particular structures on plants, the galls, in which the larvae feed and develops to maturity [41]. With roughly 1400 described species, Cynipidae represents the second largest radiation of gall-inducing insects after gall midges (Diptera: Cecidomyiidae) [42]. Galls induced by gall-wasps are morphologically complex and provide shelter and nutrition for their larvae, as well as protection from predators and parasitoids [43], [44]. Notably, species of Cynipini, and a few species of Pediaspidini, have complex, cyclically parthenogenetic (heterogonic) life cycles (i.e. alternation of sexual and asexual generations), which in some cases also involve host plant alternation (heteroecy) [45]. The cynipid inquilines also have phytophagous (or maybe parasitoid in a few cases) larvae but cannot initiate gall formation on their own. Instead, their larvae develop inside the galls induced by other gall-wasps [46]. We here used the term “gall-wasps” for the family Cynipidae as a taxonomic defined group, the term “gall-inducer” for the cynipid species which induce galls (all tribes except Synergini and Paraulacini), and the term “gall-inquilines” for the cynipid and figitid species which usurp galls to feed on plant tissue (Synergini), on host larvae (Parnipinae), or on plant tissue and/or host larvae (Paraulacini, Plectocynipinae).

Data on antennal morphology, and particularly antennal sensilla in Cynipoidea, are very scarce. For males, a detailed comparative study analysed the morphology of a particular antennal gland believed to release and spread sexual pheromones [47] (and thus did not concern sensilla). For females, detailed studies on antennal sensilla are available only for three species of Figitidae in a single subfamiliy (Eucoilinae) [48], [49] and for only 1 species of Cynipidae (Cynipini) [50]. In addition, one single type of sensilla (sensilla placoidea) was studied in 9 species of Cynipoidea within the large order-level comparative work of Basibuyuk and Quicke [51] devoted to this sensilla type. We here greatly extend such information to all the eight described cynipid tribes [52] as well as the main genera of Cynipidae; to this, we add new species of Figitidae belonging to not yet explored subfamilies, and provide the first data on Ibaliidae and Liopteridae. This allows detailed comparison of antennal morphology and sensillar equipment among many species and development of preliminary hypotheses on their evolution by mapping of salient traits onto the most recent cynipoid phylogeny. We then look for possible associations between such traits and life-history traits such as foraging strategy and plant resource use.

## Materials and Methods

### Selected taxa

Females of 41 species of Cynipidae, eight species of Figitidae (five parasitoids not associated with galls, two that are gall-parasitoids and one whose biology is unknown but that is closely related to a gall-parasitoid species), one species of Liopteridae and one species of Ibaliidae (both parasitoids not associated with galls) were investigated (Table 1). For heterogonic cynipid species, either sexual or asexual forms (both forms for two species) were used. The few species of Figitidae (though spanning most of the extant subfamilies) and the two species of basal Cynipoidea were included here as a sort of “outgroup” to facilitate hypotheses on antennal and sensillar evolution in gall-wasps, which are the focus of the present work. The studied gall-wasp taxa were selected to represent, on one hand, all the main lineages of gall-inducers (Cynipidae) spanning a wide range of biologies (e.g. plant type, gall structure) and, on the other hand, the taxonomic and biological (host) diversity of gall-inquilines (Table 1). The antennae of one to three females per species were analyzed morphologically. Voucher specimens are deposited at Museo Nacional de Ciencias Naturales (CSIC) (Madrid, Spain).

**Table 1:** Classification, biology and collection site for the cynipoid species included in the study.

Taxon	Biology	Collection country
Cynipoidea: Cynipidae: Aylacini		
*Aulacidea freesei* Nieves-Aldrey 1994 (2)	Galler on *Silybum* (Asteraceae)	Spain
*Aulacidea tragopogonis* (Thomson 1877) (2)	Galler on *Tragopogon* (Asteraceae)	Spain
*Aylax papaveris* (Perris 1839) (2)	Galler on *Papaver* (Papaveraceae)	Spain
*Diastrophus rubi* (Bouche 1834) (3)	Galler on *Rubus* (Rosaceae)	Spain
*Hedickiana levantina* (Hedicke 1928) (1)	Galler on *Salvia* (Lamiaceae)	Jordan
*Iraella luteipes* (Thomson 1877) (2)	Galler on *Papaver* (Papaveraceae)	Spain
*Isocolus lichtensteini* (Mayr 1882) (2)	Galler on *Centaurea* (Asteraceae)	Spain
*Liposthenes kerneri* (Wachtl 1891) (1)	Galler on *Nepeta* (Lamiaceae)	Spain
*Panteliella fedtschenkoi* (Rubsaamen 1896) (1)	Galler on *Phlomis* (Lamiaceae)	Romania
*Phanacis centaureae* Förster 1860 (1)	Galler on *Centaurea* (Asteraceae)	Spain
*Timaspis phoenixopodos* Mayr 1882 (1)	Galler on *Lactuca* (Asteraceae)	Spain
*Xestophanes potentillae* (Retzius in De Geer 1773) (1)	Galler on *Potentilla* (Rosaceae)	Spain
Cynipoidea: Cynipidae: Cynipini		
*Andricus burgundus* Giraud 1859 (S) (3)	Galler on *Quercus* (Fagaceae)	Spain
*Andricus coriarius* (Hartig 1843) (A) (2)	Galler on *Quercus* (Fagaceae)	Spain
*Andricus crispator* Tschek 1871 (S) (2)	Galler on *Quercus* (Fagaceae)	Spain
*Andricus curvator* Hartig 1840 (S) (2)	Galler on *Quercus* (Fagaceae)	Spain
*Andricus grossulariae* Giraud 1859 (A) (3)	Galler on *Quercus* (Fagaceae)	Spain
*Andricus grossulariae* Giraud 1859 (S) (2)	Galler on *Quercus* (Fagaceae)	Spain
*Andricus multiplicatus* Giraud 1859 (S) (2)	Galler on *Quercus* (Fagaceae)	Hungary
*Andricus pictus* (Hartig 1856) (A) (3)	Galler on *Quercus* (Fagaceae)	Spain
*Andricus quercusradicis* (Fabricius 1798) (A) (2)	Galler on *Quercus* (Fagaceae)	Spain
*Andricus quercusradicis* (Fabricius 1798) (S) (1)	Galler on *Quercus* (Fagaceae)	Spain
*Andricus quercusramuli* (Linnaeus 1761) (S) (2)	Galler on *Quercus* (Fagaceae)	Spain
*Cynips quercusfolii* Linnaeus 1758 (A) (2)	Galler on *Quercus* (Fagaceae)	Spain
*Dryocosmus kuriphilus* Yasumatsu 1951 (1)	Galler on *Castanea* (Fagaceae)	Italy
*Plagiotrochus quercusilicis* (Fabricius 1798) (S) (1)	Galler on *Quercus* (Fagaceae)	Spain
*Pseudoneuroterus macropterus* (Hartig 1843) (A) (2)	Galler on *Quercus* (Fagaceae)	Hungary
*Trigonaspis mendesi* Tavares 1902 (A) (1)	Galler on *Quercus* (Fagaceae)	Spain
*Trigonaspis synaspis* (Hartig 1841) (S) (2)	Galler on *Quercus* (Fagaceae)	Spain
Cynipoidea: Cynipidae: Diplolepidini		
*Diplolepis rosae* (Linnaeus 1758) (2)	Galler on *Rosa* (Rosaceae)	Spain
Cynipoidea: Cynipidae: Eschatocerini		
*Eschatocerus acaciae* Mayr 1881 (2)	Galler on *Prosopis* and *Acacia* (Fabaceae)	Argentina
Cynipoidea: Cynipidae: Paraulacini		
*Cecinothofagus gallaelenga* Nieves-Aldrey & Liljeblad 2009 (1)	Gall-parasitoid or gall-inquiline of *Aditrochus* (Chalcidoidea) galls on *Nothofagus*	Chile
Cynipoidea: Cynipidae: Pediaspidini		
*Pediaspis aceris* (Gmelin 1790) (asexual) (1)	Galler on *Acer* (Sapindaceae)	Spain
Cynipoidea: Cynipidae: Qwaqwaiini		
*Qwaqwaia scolopiae* Liljeblad, Nieves-Aldrey & Melika 2011 (1)	Galler on *Scolopia* (Salicaceae)	South Africa
Cynipoidea: Cynipidae: Synergini		
*Ceroptres cerri* Mayr 1873 (1)	Gall-inquiline of *Plagiotrochus* + other Cynipini	Spain
*Periclistus brandtii* (Ratzeburg 1832) (1)	Gall-inquiline of *Diplolepis*	Spain
*Rhoophilus loewi* Mayr 1881 (1)	Gall-inquiline of *Scyrotis* (Lepidoptera: Cecidosidae)	South Africa
*Saphonecrus lusitanicus* (Tavares 1901) (1)	Gall-inquiline of *Andricus* + *Plagiotrochus*	Spain
*Synergus clandestinus* Weld 1952 (1)	Gall-inquiline of *Andricus*	Spain
*Synergus hayneanus* (Ratzeburg 1833) (1)	Gall-inquiline of *Andricus*	Spain
*Synergus physocerus* Hartig 1843 (1)	Gall-inquiline of *Trigonaspis*	Spain
*Synergus umbraculus* (Olivier 1791) (2)	Gall-inquiline of *Andricus*	Spain
*Synophrus politus* Hartig 1843 (2)	Gall-inquiline of *Andricus*	Spain
Cynipoidea: Figitidae: Anacharitinae		
*Acanthaegilips* sp. (1)	Endoparasitoid of Neuroptera: Chrysopidae and Hemerobiidae (unconcealed)	Colombia
Cynipoidea: Figitidae: Aspicerinae		
*Callaspidia notata* (Boyer de Fonsc., 1832) (1)	Endoparasitoid of Diptera: Cyclorrhapha (unconcealed)	Spain
Cynipoidea: Figitidae: Charipinae		
*Apocharips* sp. (1)	Endoparasitoid of Hymenoptera: Braconidae and Chalcidoidea (unconcealed)	Spain
Cynipoidea: Figitidae: Eucolinae		
*Ganaspis* sp. (1)	Endoparasitoid of Diptera: Cyclorrhapha (unconcealed)	Spain
Cynipoidea: Figitidae: Figitinae		
*Neralsia* sp. (1)	Endoparasitoid of Diptera: Cyclorrhapha (unconcealed)	Colombia
Cynipoidea: Figitidae: Parnipinae		
*Parnips nigripes* (Barbotin 1963) (1)	Gall-parasitoid of *Barbotinia* (Aylacini) in *Papaver*	Spain
Cynipoidea: Figitidae: Plectocynipinae		
*Araucocynips queulensis* (Buffington & Nieves-Aldrey 2011) (1)	Biology unknown. In *Nothofagus* forests	Chile
*Plectocynips pilosus* (Ros-Farre 2002) (1)	Gall-parasitoid or gall-inquiline of *Aditrochus* on *Nothofagus*	Chile
Cynipoidea: Ibaliidae: Ibaliinae		
*Ibalia rufipes* Cresson 1879	Endoparasitoid of Hymenoptera: Siricidae (in wood)	Spain
Cynipoidea: Liopteridae: Oberthuerellinae		
*Oberthuerella* sp.	Unknown, but likely endoparasitoids of wood-boring insects (Coleoptera: Buprestidae; Hymenoptera: Siricidae)	Cameroon

The number of individuals studied is in brackets. S = sexual generation, A = asexual generation. Depository: JLNA — J. L. Nieves-Aldrey collection, Museo Nacional de Ciencias Naturales, Madrid.

For all species except three collected in Chile no specific permissions were required for the locations/activities, since collections were done in non-protected areas. The three species from Chile were collected in the Reserva Nacional Los Queules, and the permit for such collection was issued by the Corporación Nacional Forestal (CONAF). The field studies did not involve endangered or protected species.

### Scanning electron microscopy (SEM)

Females were dissected under light microscopy and the excised antennae were gold-coated after mounting on adhesive carbon pads attached to aluminium stubs. For the few specimens coming from the Museum collection, we introduce into the SEM the whole, not gold-coated, individuals.

The sensilla on antennae were studied by analyzing SEM images obtained using an ESEM QUANTA 200 microscope (FEI Company, Oregon-USA) at the Museo Nacional de Ciencias Naturales (Madrid, Spain). High vacuum conditions (resolution: 3.0 nm at 30 kV (SE), 10 nm at 3 kV (SE), and 4.0 nm at 30 kV (BSE)) were used on previously gold-coated samples. The accelerating voltage was 26 kV, the high vacuum was 0.40–0.50 torr, and the working distance was 10 mm. Antennae were observed in dorsal, ventral and lateral view.

The number of sensilla was not recorded exactly on each segment due to the orientation of some antenna. However, we counted all sensilla in certain well visible segments. The sample of sensilla used for size calculations varies among individuals and species, depending on their visibility/definition in the SEM images (1–3 sensilla per type). Because of the small sample size (number of individuals and antennae), we give numerical results as ranges rather than means. We calculated lengths and widths of flagellomeres and sensilla from pictures taken at adequate magnifications (up to 300×), importing them into ImageJ software (National Institutes of Health, USA), where calculations were made.

High-resolution SEM digital images of antennae and sensilla types of the studied species will be deposited in MorphoBank (www.morphobank.org).

### Terminology

For general antennal morphology we referred to the well-established classification for Hymenoptera, mostly based on shape patterns [53]. Antennae are composed of (proximally to distally) a scape, a pedicel, and a number of antennal segments (flagellomeres) jointly called the flagellum. They can be as either filiform (as wide proximally and distally, not or weakly expanding distally), or clavate (strongly expanded distally). Filiform antennae can be either linear and slender or so-called moniliform (i.e., like a string of beads). The flagellomeres were designated F_1_ to F_A_ (distal flagellomere, that could be F_10_ to F_13_, see Results), in a proximal to distal direction, with F_n_ designing the flagellomere just before F_A_. For the sensilla inventory, we primarily followed the classification of sensilla by Callahan [54], Walther [12], van Baaren et al. [11] and Romani et al. [55], based on morphological characters. We also refer to definitions found in The Hymenoptera Anatomy Ontology (HAO) project portal [56], [57] and highlight in the Discussion where HAO definitions should be implemented following our descriptions of sensilla, or where a new terminology should be added to the HAO project. The classification here used, however, should be considered, for some sensilla types, as preliminary because the internal structure and function of different types of sensilla are not yet fully known [58].

Following these references and further previous studies on Hymenoptera (see below), we defined the different types of sensilla as follows: Sensilla placoidea (SP) were defined as multiporous, elongate, plate-like sensilla with a large surface area [51]. Sensilla coeloconica (SCo) are defined as poreless sensilla with a cuticular peg standing on the antennal surface and presenting a “collar” of wrinkled cuticle surrounding the peg [59]. Sensilla campaniformia (SCa) are defined as poreless, button-like knob sensilla emerging from an opening in the centre of a circular cuticular disk, thus protected in small depressions on the surface of the cuticle [30]. The sensilla basiconica (SB) are multi-porous sensilla with a typical stout, cylindrical, variably bulbous morphology [60]. Sensilla trichoidea (ST) include aporous, single-or multi-pore hair-like structures ending in a tip; they can be short to long, curved or straight, and may or may not feature longitudinal furrows [55]. The detailed descriptions of all types of sensilla found in our studied species are presented in the Results.

### Morphological characters

Due to the small number of individuals, and thus antennae, analysed per species, it was not possible to study in detail the distribution of all sensilla types along the whole flagellum. However, previous studies in Hymenoptera agree that both sensilla types and sensilla numbers significantly increase from proximal to distal flagellomeres (e.g. [33] and references therein), so that for the investigation of sensilla morphology, distribution and density, we here largely refer to the last funicular flagellomere (F_n_) + the apical flagellomere (F_A_). Preliminary observation of the whole antennae in a few species confirmed the proximal-distal increasing trend in Cynipoidea. However, for a couple of large types of sensilla (SP and SCo) it was possible to study the presence and arrangement along the whole antenna. For the other types of sensilla, we only described the morphology and reported their occurrence. If one type of sensilla was not found in F_n_ – F_A_, we then checked the other flagellomeres in order to ascertain if the species does or doesn't have such sensilla in the antennae as a whole.

To explore the relationships among species based on the presence/absence of the different sensillar types in the antennae, we carried out a hierarchical cluster analysis, which finds relatively homogeneous clusters of cases based on the chosen variables. The cluster analysis was performed using Ward's method based on Euclidean distances (dissimilarity) between pairs of objects [33]; this analysis also reported the dissimilarity value (truncation), which likely determines how many clusters best suit the data.

Overall, the morphological component of this study includes 35 characters. The first five characters are based on differences in shape, relative size and number of antennal segments. The remaining 30 characters are based on differences in shape, relative size, occurrence and number of the different sensilla types. Some characters are not applicable to all species and these were coded as missing data (−) (Table 2). Some cases of multistate characters were also included in the final data matrix (Table 2). With the exception of the characters concerning only the absence or presence of the different sensilla types, the characters' states are visually represented in the Figs. S1, S2, S3, S4. In the text, characters are referenced in the form, e.g. “21-1”, where “21” is the character and “1” the character state.

**Table 2:** Data matrix based on the characters listed in the Materials and methods.

	Characters																																		
Taxon	1	2	3	4	5	6	7	8	9	10	11	12	13	14	15	16	17	18	19	20	21	22	23	24	25	26	27	28	29	30	31	32	33	34	35
*Acanthaegilips* sp.	1	1	0	0	1	5	0	1	1	1	1	1	1	0	1	0	0	0	0	1	1	1	0	0	0	1	1	0	1	0	0	0	0	1	3
*Andricus burgundus* (S)	1	0	1	–	0	8	0	0	0	1	0	0	0	0	0	0	1	0	1	1	1	0	1	1	1	1	1	1	1	0	0	0	0	0	1
*Andricus coriarius* (A)	1	0	0	0	0	8	0	0	0	0	0	0	1	0	0	0	1	0	1	1	1	0	0	1	1	1	1	1	1	0	0	0	0	1	2
*Andricus crispator* (S)	0	1	0	0	0	8	0	0	0	0	0	0	0	0	0	0	1	0	1	1	1	0	1	1	1	1	1	1	1	0	0	0	0	0	1
*Andricus curvator* (S)	1	0	0	0	0	8	0	0	0	1	0	0	1	0	0	0	1	0	1	1	1	0	2	1	1	1	1	1	1	0	0	0	0	0	1
*Andricus grossulariae* (A)	1	0	1	–	0	8	0	0	1	1	0	0	1	1	0	0	0/1	0	0	1	1	0	1	1	1	1	1	1	1	0	0	0	0	1	1
*Andricus grossulariae* (S)	0	1	0	0	0	8	0	0	1	1	0	0	1	0	0	0	1	0	1	1	1	0	1	1	1	1	1	1	1	0	0	0	0	1	1
*Andricus multiplicatus* (S)	1	0	0	0	0	8	0	0	0	1	0	0	1	1	0	1	1	0	1	1	1	0	1	1	1	1	1	1	1	0	0	0	0	1	1
*Andricus pictus* (A)	2	1	0	0	0	8	0	0	1	1	0	0	1	0	0	0	1	0	1	0	1	0	2	1	1	1	1	1	1	0	0	0	0	1	1
*Andricus quercusradicis* (A)	1	2	0	0	0	8	0	0	1	1	0	0	1	1	0	0	1	0	1	1	2	0	0	1	1	1	1	1	1	0	0	0	0	0	2
*Andricus quercusradicis* (S)	1	1	0	0	0	8	0	0	0	0	0	0	0	0	0	0	1	0	1	0	1	0	2	1	1	1	1	1	1	0	0	0	0	0	1
*Andricus quercusramuli* (S)	1	1	1	–	0	8	0	0	1	1	0	0	1	0	0	0	1	0	1	1	1	0	2	1	1	1	1	1	1	0	0	0	0	0	1
*Apocharips* sp.	1	0	1	–	1	7	0	0	0	1	0	1	0	0	0	0	1	0	–	0	1	0	0	1	1	1	1	1	1	0	0	0	0	0	1
*Araucocynips queulensis*	1	0	2	–	0	7	0	0	0	1	0	1	1	0	0	0	0	1	1	1	1	1	0	0	0	0	1	1	1	1	0	1	0	0	2
*Aulacidea freesei*	0	0	0	0	1	8	0	0	0	1	0	1	0	0	0	0	0	0	1	1	1	1	0	0	1	1	1	1	1	1	0	0	0	0	2
*Aulacidea tragopogonis*	1	1	0	0	1	9	0	1	1	1	0	1	0	1	0	0	0	0	1	1	1	1	1	1	1	1	1	1	1	1	0	0	0	0	1
*Aylax papaveris*	2	0	0	0	1	8	0	1	1	1	0	1	1	1	0	0	0	0	1	1	1	0	1	1	1	1	1	1	1	0	0	0	0	0	1
*Callaspidia notata*	1	1	0	0	1	7	0	1	1	1	1	1	1	0	0	0	1	1	0	1	1	1	0	0	1	1	1	0	1	0	1	0	0	0	3
*Cecinothofagus gallaelenga*	0	0	2	–	0	9	0	0	0	0	0	1	1	0	0	1	0	1	–	0	0	–	–	1	1	1	1	1	1	1	0	0	1	1	1
*Ceroptres cerri*	0	0	1	–	1	7	0	0	0	1	0	1	1	0	0	0	1	0	1	1	2	0	1	1	1	1	1	0	1	0	0	0	0	0	1
*Cynips quercusfolii* (A)	1	1	0	0	0	8	0	0	0	0	0	0	1	1	0	0	1	0	1	1	1	0	0	1	1	1	1	1	1	0	0	0	0	2	1
*Diastrophus rubi*	1	1	0	0	1	8	1	1	1	1	0	1	1	0	0	0	1	1	1	1	1	1	1	1	1	1	1	1	1	0	0	0	0	0	1
*Diplolepis rosae*	2	2	0	0	1	6	0	1	1	0	1	1	1	0	0	0	1	1	0	1	1	1	0	0	0	1	1	1	1	0	0	0	0	0	1
*Dryocosmus kuriphilus* (A)	2	1	0	0	0	8	1	1	1	1	0	1	0	0	0	0	1	1	1	1	1	0	2	1	1	1	1	1	1	0	0	0	0	0	1
*Eschatocerus acaciae*	1	1	0	0	1	6	0	1	1	1	0	1	1	1	0	0	0	0	1	1	2	1	2	1	1	0	1	0	1	0	0	0	0	0	0
*Ganaspis* sp.	1	0	0	1	1	4	0	0	0	0	0	1	0	0	0	0	0	0	1	1	1	1	0	0	0	0	0	1	1	0	0	0	0	0	0
*Hedickiana levantina*	0	0	0	0	1	7	0	0	1	1	1	1	2	1	0	0	0	0/1	0	1	2	1	0	1	1	1	1	0	1	0	0	0	0	0	2
*Ibalia rufipes*	1	0	0	0	1	6	0	1	1	1	2	1	1	1	0	0	0	1	–	1	0	–	–	0	1	1	1	0	1	0	0	0	0	0	2
*Iraella luteipes*	2	0	0	0	1	8	0	1	1	1	0	1	0	0	0	0	0	0	1	1	1	1	0	1	1	1	1	1	1	0	0	0	0	0	2
*Isocolus lichtensteini*	0	0	0	0	1	9	1	1	1	1	1	1	1	1	0	0	0	0	0	1	1	1	0	1	1	1	1	1	1	1	0	0	0	0	2
*Liposthenes kerneri*	0	0	0	0	1	8	1	1	1	1	0	1	0	0	0	0	1	0	1	1	1	1	0	1	1	0	1	1	1	1	0	0	0	0	1
*Neralsia* sp.	1	0	0	1	0	9	0	1	1	1	1	1	1	2	0	0	0	0	1	1	1	0	0	1	1	1	1	1	1	1	0	0	0	0	0
*Oberthuerella sp.*	1	1	1	–	0	5	0	1	1	1	2	1	2	2	0	0	0	0	–	0	0	–	–	0	0	1	1	0	1	0	1	0	0	1	3
*Panteliella fedtschenkoi*	2	0	0	0	1	7	0	0	1	1	0	1	0	1	0	0	0	0	1	1	1	1	0	0	1	1	1	1	1	0	0	0	0	0	1
*Parnips nigripes*	1	0	0	0	1	8	1	1	1	1	1	1	1	1	0	0	1	0	0	1	1	0	0	1	1	1	1	1	1	0	0	0	0	0	2
*Pediaspis aceris* (A)	3	1	0	0	0	8	0	1	1	1	2	1	1	1	0	0	0	1	0	1	3	0	1	1	1	1	1	1	1	0	0	0	0	1	1
*Periclistus brandtii*	0	0	0	0	1	9	0	1	1	1	1	1	0	0	0	0	1	0/1	1	1	1	0	0	1	1	1	1	1	1	1	0	0	0	0	2
*Phanacis centaureae*	2	2	0	0	1	9	0	0	1	1	0	1	0	0	0	0	0	0	1	0	1	1	0	1	1	1	1	1	1	0	1	0	0	0	1
*Plagiotrochus quercusilicis* (S)	0	0	0	0	1	7	0	0	0	1	0	1	0	0	0	0	1	0/1	1	1	1	0	2	0	1	1	1	1	1	0	0	0	0	0	1
*Plectocynips pilosus*	1	0	2	–	0	6	0	1	1	1	0	1	1	0	0	0	1	0	0	0	2	1	0	0	0	0	1	1	1	0	0	1	0	0	1
*Pseudoneuroterus macropterus* (A)	0	0	0	0	1	9	0	0	1	1	0	1	2	1	0	0	1	0	1	1	2	0	0	1	1	1	1	1	1	1	0	0	0	0	1
*Qwaqwaia scolopiae*	2	0	0	0	0	7	0	1	1	1	1	1	1	1	0	0	1	1	–	0	0	–	–	1	1	1	1	1	1	0	0	0	0	0	1
*Rhoophilus loewi*	2	1	0	0	1	7	1	1	1	1	0	1	1	0	0	1	1	0	–	0	0	–	–	1	1	1	1	1	1	0	0	0	0	0	1
*Saphonecrus lusitanicus*	1	1	0	0	1	6	0	1	1	1	0	1	1	0	0	0	1	0	–	1	1	0	1	0	0	1	1	1	1	0	0	0	0	0	2
*Synergus clandestinus*	2	2	0	0	1	5	0	0	0	0	0	0	1	0	0	0	1	0	–	0	0	–	–	0	0	1	1	1	1	0	0	0	0	0	2
*Synergus hayneanus*	2	0	0	0	1	10	0	0	0	0	0	1	0	0	0	0	0	0	1	1	1	0	1	1	1	1	1	1	1	1	1	0	0	0	2
*Synergus physocerus*	2	2	0	0	1	8	0	0	0	0	1	1	0	0	0	1	1	0/1	1	0	1	0	0	1	1	1	1	1	1	0	0	0	0	0	2
*Synergus umbraculus*	2	0	0	0	1	5	0	0	0	1	1	1	0	0	0	0	1	0	–	0	0	–	–	0	0	1	1	1	1	0	0	0	0	0	2
*Synophrus politus*	1	0	0	0	1	8	0	0	0	1	1	1	2	2	0	0	1	0	0	1	1	0	0	1	1	1	1	1	1	0	0	0	0	0	2
*Timaspis phoenixopodos*	3	2	0	0	1	9	0	1	1	1	1	1	0	1	0	0	0	0	1	1	1	1	0	1	1	1	1	1	1	1	0	0	0	0	1
*Trigonaspis mendesi* (A)	1	2	0	0	0	9	0	0	0	1	0	0	1	0	0	0	1	1	1	1	1	0	0	1	1	1	1	1	1	1	0	0	0	0	1
*Trigonaspis synaspis* (S)	2	1	0	0	1	7	0	1	1	1	0	1	0	0	0	1	1	1	1	1	1	0	0	0	1	1	1	1	1	0	0	0	0	1	2
*Xestophanes potentillae*	1	0	0	0	1	9	0	0	1	1	0	1	0	0	0	0	0	0	1	1	1	1	0	1	1	1	1	1	1	0	1	0	0	0	1

“–” was used to denote a character that is not applicable to that species; multi-state characters (0/1) occurred in some species.

The characters and character states are described below (the full description of each sensilla type is lined in the Results). Characters are specific to female Cynipoidea.

1. Number of completely separated flagellomeres: (0) 10; (1) 11; (2) 12; (3) 13 (Fig. S1)
2. Length of F_1_: (0) short, about as long as F_2_ (range 0–1.1); (1) 1.2–1.5 longer than F_2_; (2) >1.5 longer than F_2_ (range 1.5–2) (Fig. S1)
3. Shape of antennal flagellum: (0) filiform; (1) slightly expanded from base to apex; (2) clavate (Fig. S1)
4. Shape of filiform flagellum: (0) linear and slender; (1) moniliform (Fig. S1)
5. Relative length of F_n_. (0) as wide as long; (1) clearly longer than wide (Fig. S1)
6. Total number of sensilla types observed on F_n_ - F_A_: actual number (range: 4–10)
7. Sensilla placoidea (SP) on F_1_: (0) absent; (1) present
8. Sensilla placoidea (SP) on F_2_: (0) absent; (1) present
9. Sensilla placoidea (SP) on F_3_: (0) absent; (1) present
10. Sensilla placoidea (SP) on F_4_: (0) absent; (1) present
11. Arrangement of sensilla placoidea (SP) on F_n_: (0) arranged in one row (1) two rows (2) three-four rows (Fig. S2)
12. Arrangement of sensilla placoidea (SP) on F_n_: (0) present only dorso-laterally (1) present on the whole surface (Fig. S2)
13. Number of sensilla placoidea (SP) visible in each row: (0) 3–5; (1) 6–8; (2) >8 (Fig. S2)
14. Relative separation of sensilla placoidea (SP) on a row: (0) widely separated (> as width of a sensillum) (1) narrowly separated (< as width of a sensillum); (2) closely spaced, almost contiguous (Fig. S2)
15. Shape of sensilla placoidea (SP): (0) almost flat, only slightly or not rising on the segment; (1) ridge-like, clearly raising on the segment (Fig. S2)
16. Surface of sensilla placoidea (SP): (0) always simple; (1) at least some with a distinct longitudinal groove (Fig. S2)
17. Relative extension of the sensilla placoidea (SP): (0) at most only reaching the distal margin of segment; (1) more or less overlapping the distal margin of segment (Fig. S2)
18. Shape of a sensilla placoidea (SP): (0) linear, with parallel margins; (1) more or less sinuate (Fig. S2)
19. Presence of sensilla coeloconica type A (SCo-A) in the flagellum (up to F_n_): (0) From the proximal part of flagellum (F_2_-F_4_ to F_n_); (1) from the middle part of flagellum (F_5_-F_8_ to F_n_) (Fig. S3)
20. Sensilla coeloconica type A (SCo-A) in F_A_: (0) absent; (1) present (Fig. S3)
21. Maximum number of sensilla coeloconica type A (SCo-A) in a flagellomere: (0) absent, (1) 1; (2) 2; ≥3 (3) (Fig. S3)
22. Relative position of sensilla coeloconica type A (SCo-A) on a flagellomere: (0) on or close the distal margin, (1) far from the distal margin (Fig. S3)
23. Relative size of the pit of sensilla coeloconica type A (SCo-A) (compared with width of F_n_): (0) small (range 0.03–0.05), (1) medium size (range 0.06–0.08); (2) large (range 0.10–0.12) (Fig. S3)
24. Sensilla coeloconica type B (SCo-B): (0) absent; (1) present
25. Sensilla campaniformia (SCa): (0) absent; (1) present
26. Sensilla basiconica (SB): (0) absent; (1) present
27. Sensilla trichoidea type A (ST-A): (0) absent; (1) present
28. Sensilla trichoidea type B (ST-B): (0) absent; (1) present
29. Sensilla trichoidea type C (ST-C): (0) absent; (1) present
30. Sensilla trichoidea type D (ST-D): (0) absent; (1) present
31. Sensilla trichoidea type E (ST-E): (0) absent; (1) present
32. Large disc sensilla (SLD): (0) absent; (1) present
33. Large volcano sensilla (SLV): (0) absent; (1) present
34. Length of sensilla trichoidea (all types together) on F_1_ related to F_A_: (0) similar; (1) slightly different; (2) strongly different (Fig. S4)
35. Number of sensilla trichoidea (all types together) on F_n_, measured in a row along its length: (0) very few (1–2), (1) some (4–9); (2) many (10–15); (3) very dense and abundant (>15) (Fig. S4)

### Phylogenetic trait mapping

**Figure 1:**
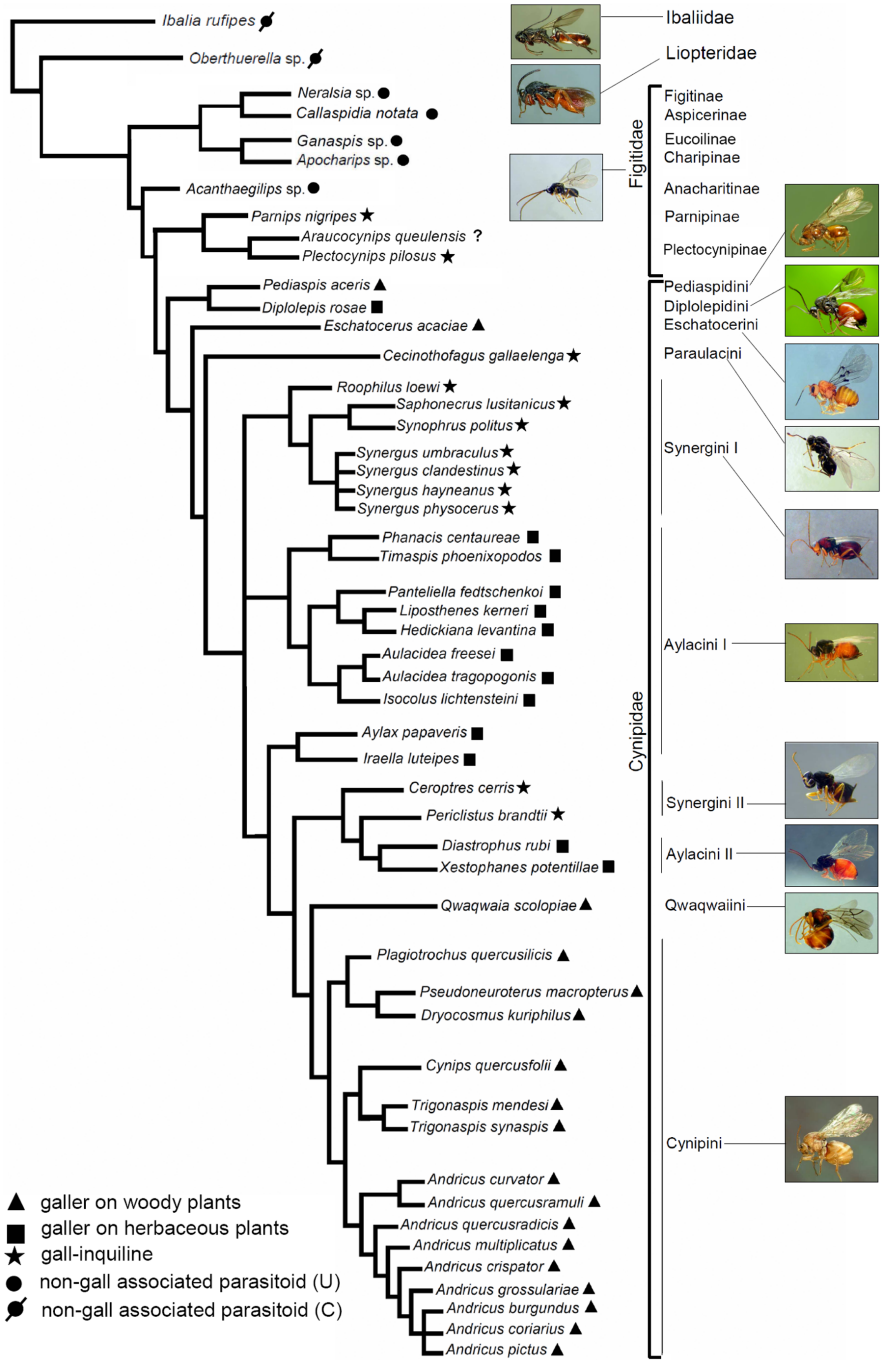
Phylogenetic relationships among the studied species of Cynipoidea, as depicted from recent studies [59]–[64] and unpublished data (see Methods for details). The main life-history trait for each species is mapped on the tree. For non-gall parasitoids, U = unconcealed host and C = concealed host. “?” denotes that biology is unknown for *Araucocynips queulensis*.

In an attempt to map our results onto a phylogeny of the studied species, we generated an intuitive phylogenetic tree based on combined molecular and morphological phylogenetic analyses available in recent works [61]–[66] and more recent unpublished results obtained in an on-going study in which one of the authors of the present paper (JLN-A) is involved (Fig. 1). This tree was first built for a recent study on the evolution of metal inclusion in mandibles and ovipositors of Cynipoidea [67] and it is modified here to include the species considered (Fig. 1). For a detailed description on how the relationships between lineages were reconstructed, we refer to Polidori et al. [67]. In summary, most information comes from a recent molecular study on Cynipidae including all the tribes except Qwaqwaiini and Paraulacini [62], from Nieves-Aldrey [65] and unpublished data (for the position of Qwaqwaiini and Paraulacini), from Stone et al. [64] and Ács et al. [66] for some genera and species of Cynipini and Synergini (Cynipidae) not included in [61], and from recent combined molecular + morphological studies [61]–[63] for the phylogenetic position of the parasitic groups of Cynipoidea (Liopteridae, Ibaliidae and Figitidae). In particular within Figitidae, however, the depicted topology should be considered as just one of the numerous, still weakly supported, scenarios obtained with different phylogenetic analyses (parsimony or Bayesian inference) and with different types of data (molecular or morphological) [61]–[62], with the most recent (unpublished) morphological+molecular analysis returning many unresolved relationships between figitid subfamilies. We arbitrarily decided here to follow the scenario hypothesized by the last published figitid phylogeny [62]; in particular we decided to follow the relationships obtained with the combined molecular + morphological analysis through the parsimony method. Despite such approximation in depicting such relationships, we feel that overall our working phylogeny provides a useful hypothesis for general appreciation of the possible links between phylogeny, antennal/sensillar morphology and life history traits.

## Results

### Antennae

**Figure 2:**
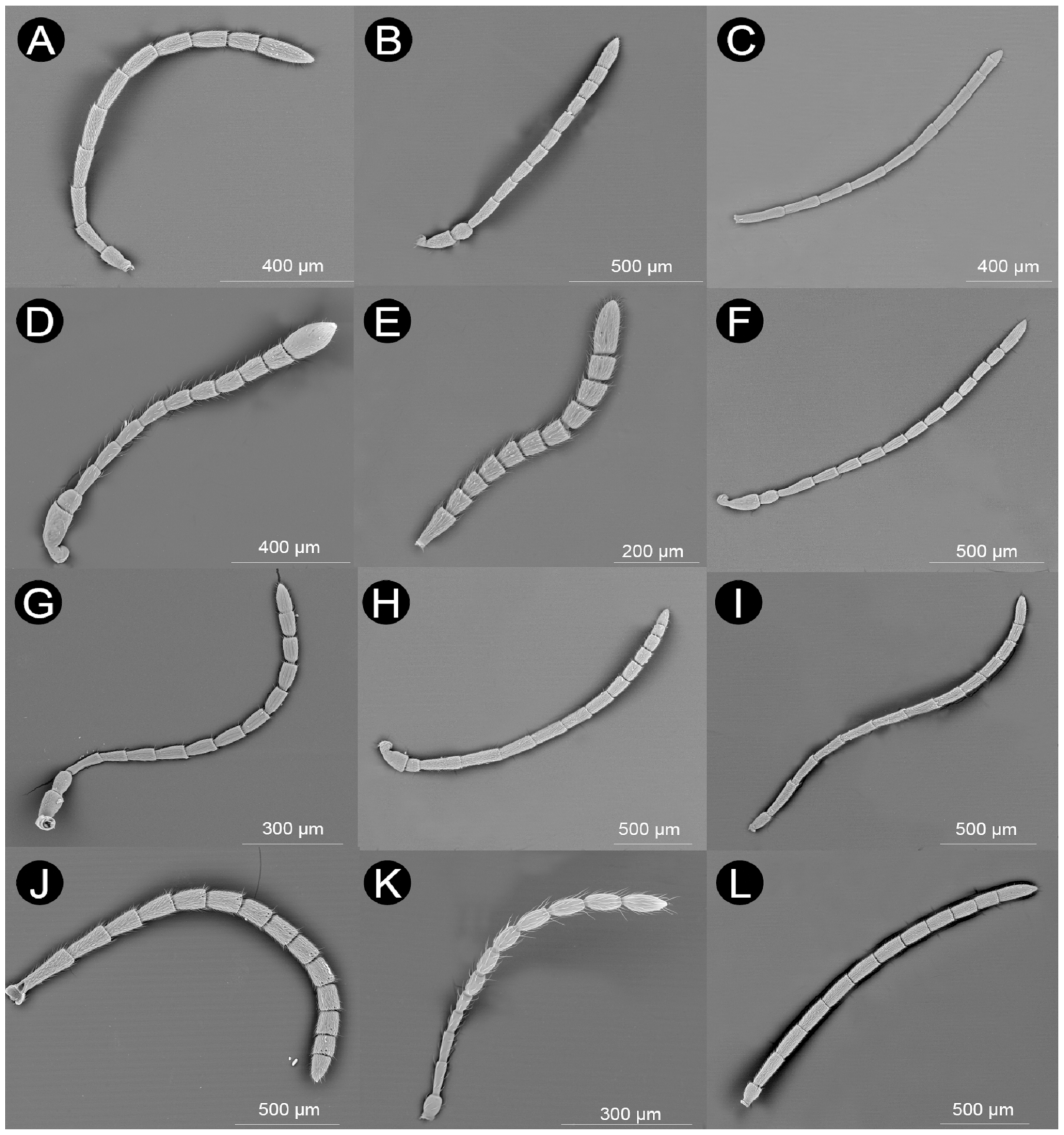
Variability in the general aspect of the antennae of Cynipoidea. A) *Aulacidea freesei* (filiform (3-0) with 10 flagellomeres (1-0) and F_1_ about as long as F_2_ (2-0)), B) *Andricus crispator* (sexual) (filiform (3-0) with 10 flagellomeres (1-0) and F_1_ 1.2–1.5 times longer than F_2_ (2-1)), C) *Eschatocerus acaciae* (filiform (3-0) with 11 flagellomeres (1-1) and F_1_ 1.2–1.5 times longer than F_2_ (2-1)), D) *Cecinothofagus gallaelenga* (clavate (3-0) with 10 flagellomeres (1-0) and F_1_ about as long as F_2_ (2-0)), E) *Trigonaspis mendesi* (asexual) (filiform (3-0) with 11 flagellomeres (1-1) and F_1_>1.5 times longer than F_2_ (2-2)), F) *Roophilus loewi* (filiform (3-0) with 12 flagellomeres (1–2) and F_1_ 1.2–1.5 times longer than F_2_ (2-1)), G) *Phanacis centaureae* (filiform (3-0) with 12 flagellomeres (1–2) and F_1_>1.5 times longer than F_2_ (2-2)), H) *Qwaqwaia scolopiae* (filiform (3-0) with 12 flagellomeres (1–2) and F_1_ about as long as F_2_ (2-0)), I) *Synergus hayneanus* (filiform (3-0) with 12 flagellomeres (1–2) and F_1_ about as long as F_2_ (2-0)), J) *Pediaspis aceris* (asexual) (filiform (3-0) with 13 flagellomeres (1–3) and F_1_ 1.2–1.5 times longer than F_2_ (2-1)), K) *Ganaspis* sp. (moniliform (3-0; 4-1) with 11 flagellomeres (1-1) and F_1_ about as long as F_2_ (2-0)), L) *Parnips nigripes* (filiform (3-0) with 11 flagellomeres (1-1) and F_1_ about as long as F_2_ (2-0)).

The antennae of female Cynipoidea consist of a scape, a pedicel and a flagellum consisting of 10 to 13 flagellomeres (character 1: Fig. 2 and Fig. S1). The number of flagellomeres is invariably 11 in the studied species from the parasitoid lineages (Ibaliidae, Liopteridae and Figitidae), while it is quite variable in the studied gall-wasps (Cynipidae). In these Cynipidae, the highest number of flagellomeres (13) was found only in the basal tribe Pediaspidini and in one single member of Aylacini I (*Timaspis phoenixopodos* Mayr). Antennae with 12 flagellomeres are found in both basal (Diplolepidini) and derived (e.g., Aylacini I, Synergini I, Cynipini) tribes. On the other hand, the Aylacini II and most Cynipini have 11 flagellomeres, while the lowest number (10) was seen in two lineages of gall-inquilines (Synergini II and Paraulacini). The variability in the number of flagellomeres is evident when considering that within Cynipini and Aylacini I, we recorded antennae with 10, 11, 12 (and in one Aylacini I 13) flagellomeres. While the species of parasitic Cynipoidea (Ibaliidae, Liopteridae and Figitidae) here studied do not present fused distal flagellomeres, the variability found in Cynipidae is likely to depend at least partially from the variability of the number of fused segments, which is a common phenomenon.

The F_n_ length ranged between about 50 µm (*Andricus quercusramuli* (L.) (sexual)) to about 330 µm (Liopteridae) and F_n_ width from about 30 µm (*Apocharips* sp.) to about 330 µm (Liopteridae) (Table 2). When taken together in an estimated area (length×width), the F_n_ is smallest in Charipinae (Figitidae) and largest in Liopteridae and Ibaliidae, i.e. the so-called macrocynipoids, with intermediate sizes spanning all the remaining lineages.

The length of F_1_ was variable among species, when considered in relation to F_2_ (character 2: Fig. 2 and Fig. S1). Basal families and Figitidae present a mix of short (i.e. about as long as F_2_) and relatively long (1.2–1.5 longer than F_2_) F_1_. Some herb-gallers (Aylacini I and II + Diplolepidini) (60% of species) seem to have short F_1_, while most wood-gallers (Cynipini + Pediaspidini + Eschatocerini) (60% of species) have long or very long F_1_ (>1.2 longer than F_2_). Also cynipid gall-inquilines (Paraulacini + Synergini I and II) have very variable F_1_, with 60% of species falling in rank 0, 20% in rank 1 and 20% in rank 2, without visible phylogenetic effects on such pattern. However, one notes that very long F_1_ (>1.5 longer than F_2_) were seen only in Cynipidae.

The flagellum has, in most cases (90%), a classical filiform shape, i.e. with all flagellomeres of roughly constant width (character state 3-0: Fig. 2 and Fig. S1). Only three species of Cynipini, one of Synergini II, one figitid, and the liopterid, have a flagellum slightly expanded from base to apex (character state 3-1: Fig. 2 and Fig. S1), while Paraulacini and Plectocynipinae, which include inquilines/parasitoids of *Aditrochus* (Chalcidoidea: Pteromalidae) galls, have a classical clavate antenna, i.e., with flagellomeres becoming suddenly wider towards the tip of the antenna (therefore affecting mainly the last flagellomere) (character state 3-2: Fig. 2 and Fig. S1); this clava is more prominent in Paraulacini (Fig. 2). These clavae are also unique in presenting particularities in sensillar equipment (see below). Within the species showing a filiform flagellum, moreover, only two figitids show a flagellum of the moniliform subtype, i.e. with round segments making the antenna like a string of beads (character state 4-1: Fig. 1 and Fig. S1).

The shape of F_n_, when estimated as the rough ratio between length and width, varied both among and within families (character 5: Fig. 2 and Fig. S1). All figitids but *Neralsia* (Figitinae) and Plectocynipinae, together with Liopteridae, have F_n_ clearly longer than wide. *Neralsia* has flagellomeres as wide as long, while Plectocynipinae are unique in having the b proximal flagellomeres longer than wide and the distal ones as wide as long. All gall-inquilines (except Plectocynipinae (see above) and Paraulacini) have F_n_ clearly longer than wide. All herb-gallers have F_n_ clearly longer than wide and 80% of wood-gallers have F_n_ as wide as long.

### Sensilla

Sensilla found on the antennae of Cynipoidea protrude from the cuticle or sometimes lie within or beneath it. Overall, we recognized 12 types of sensilla in Cynipoidea: sensilla placoidea (SP), two types of sensilla coeloconica (SCo-A, SCo-B), sensilla campaniformia (SCa), sensilla basiconica (SB), five types of sensilla trichoidea type A (ST-A, ST-B, ST-C, ST-D, ST-E), large disc sensilla (LDS) and large volcano sensilla (LVS). Not all types, however, were found on all species (range: 4–10, character 6: Fig. 2 and Fig. S1). In particular, the lowest number of sensilla types (4) was detected in *Ganaspis* sp. (Figitidae), while 10 types were observed only in the inquiline *Synergus hayneanus* (Ratzeburg) (Synergini I).

**Figure 3:**
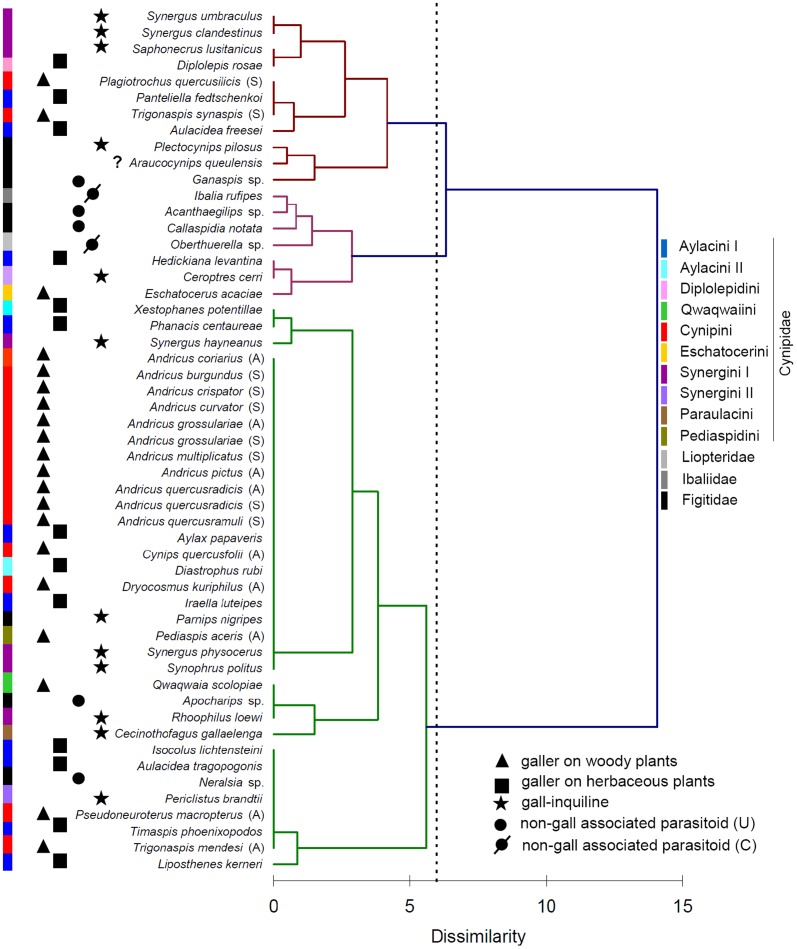
Dendrogram depicted by the Hierarchical Cluster Analysis (Ward's method) based on the matrix of presence/absence of the 12 different types of sensilla for each species. The dashed line represents the most probable truncation that segregates different clusters. The main life-history trait for each species is mapped on the dendrogram, as well as the taxonomic position of each species. For non-gall parasitoids, U = unconcealed host and C = concealed host. “?” denotes that biology is unknown for *Araucocynips queulensis*. Note that one cluster is exclusively composed of Cynipini and in particular of species in the genus *Andricus*, while the other two groups include a less defined mixture of species.

The hierarchical cluster analysis based on the presence/absence of the different types of sensilla reveals that neither the phylogenetic relationships among species nor life-history traits have any strong relationship with occurrence of sensillar types (Fig. 3). However, at least *Andricus* (Cynipini) seems homogeneous in its sensillar bouquet, and all species of this genus fall within a single sub-cluster of one of the groups proposed to be different (see truncation in Fig. 3), together with few non-Cynipini species (Fig. 3). The other two groups recognized by the statistical truncation were composed, in contrast, of a mixed assemblage of species from all the remaining lineages (Fig. 3).

The description, distribution, and occurrence of the 12 sensilla types are given in detail below.

#### Sensilla placoidea (SP)

**Figure 4:**
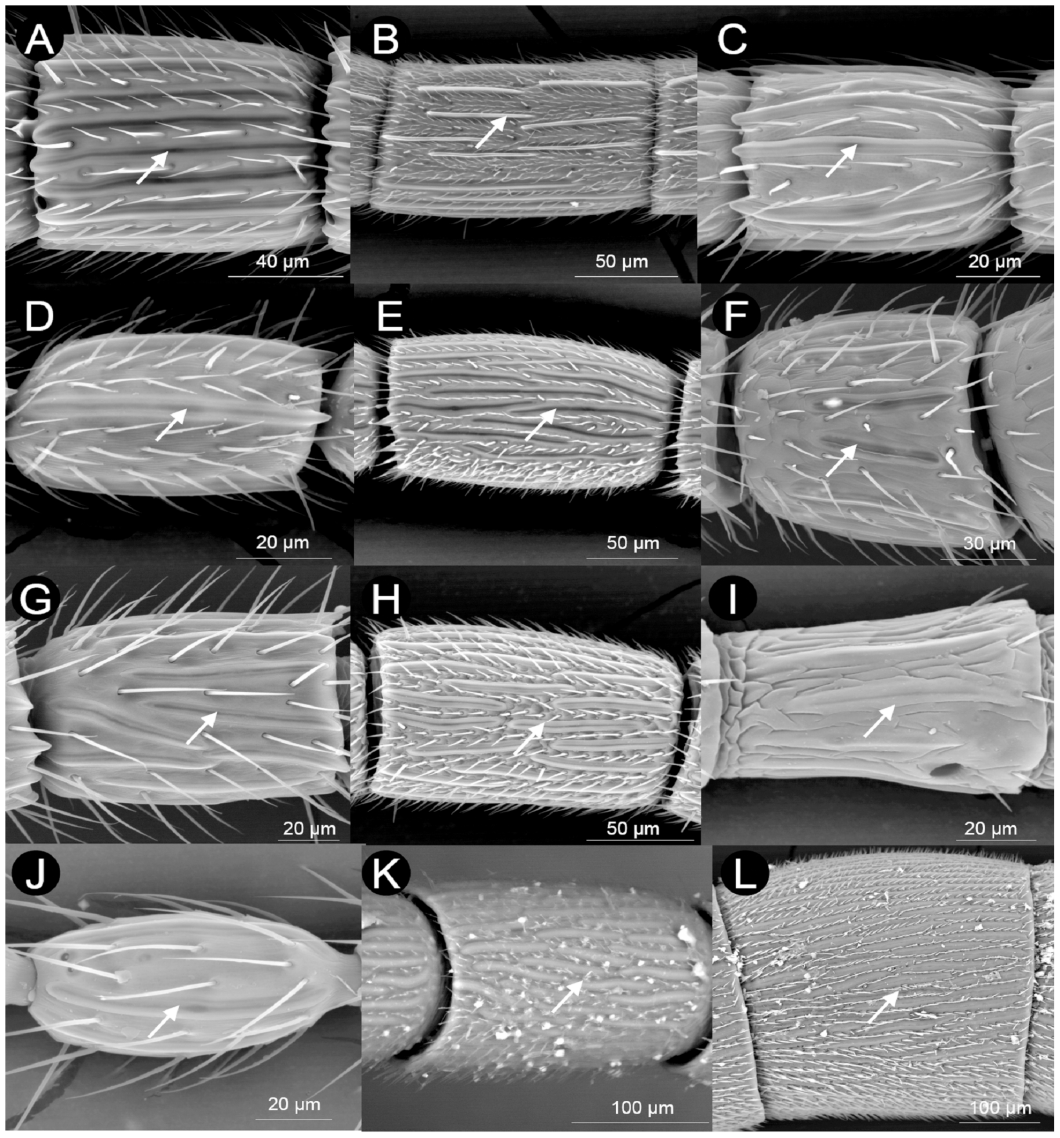
Variability in number, relative size and arrangement of sensilla placoidea (SP) in the flagellomere F_n_ of Cynipoidea. A) *Andricus corarius* (asexual) (arranged in one row (11-0), present only dorsally (12-0), 6–8 SP *per* row (13-1), widely separated in a row (14-0), almost flat (15-0), with surface constantly plane (16-0), more or less overlapping the distal margin of F_n_ (17-1), linear, with parallel margins (18-0)), B) *Acanthaegilips* sp. (arranged in two rows (11-1), present dorsally and ventrally (12-1), 6–8 SP *per* row (13-1), widely separated in a row (14-0), raising on F_n_ (15-0), with surface constantly plane (16-0), only reaching the distal margin of F_n_ (17-0), linear, with parallel margins (18-0)), C) *Andricus burgundus* (sexual) (arranged in one row (11-0), present only dorsally (12-0), 3–5 SP *per* row (13-0), widely separated in a row (14-0), almost flat (15-0), with surface constantly plane (16-0), more or less overlapping the distal margin of F_n_ (17-1), linear, with parallel margins (18-0)), D) *Apocharips* sp. (arranged in one row (11-0), present dorsally and ventrally (12-1), 3–5 SP *per* row (13-0), widely separated in a row (14-0), almost flat (15-0), with surface constantly plane (16-0), more or less overlapping the distal margin of F_n_ (17-1), linear, with parallel margins (18-0)), E) *Callaspidia notata* (arranged in two rows (11-1), present dorsally and ventrally (12-1), 6-8 SP *per* row (13-1), widely separated in a row (14-0), almost flat (15-0), with surface constantly plane (16-0), more or less overlapping the distal margin of F_n_ (17-1), more or less sinuate (18-1)), F) *Cecinothofagus gallaelenga* (arranged in one row (11-0), present dorsally and ventrally (12-1), 6–8 SP *per* row (13-1), widely separated in a row (14-0), almost flat (15-0), with a longitudinal groove (16-1), only reaching the distal margin of F_n_ (17-0), more or less sinuate (18-1)), G) *Diastrophus rubi* (arranged in one row (11-0), present dorsally and ventrally (12-1), 6–8 SP *per* row (13-1), widely separated in a row (14-0), almost flat (15-0), with surface constantly plane (16-0), more or less overlapping the distal margin of F_n_ (17-1), more or less sinuate (18-1)), H) *Hedickiana levantina* (arranged in three-four rows (11-2), present dorsally and ventrally (12-1), >8 SP *per* row (13-2), narrowly separated in a row (14-1), almost flat (15-0), with surface constantly plane (16-0), only reaching the distal margin of F_n_ (17-0)), I) *Eschatocerus acaciae* (arranged in one rows (11-0), present dorsally and ventrally (12-1), 6–8 SP *per* row (13-1), narrowly separated in a row (14-1), almost flat (15-0), with surface constantly plane (16-0), only reaching the distal margin of F_n_ (17-0), linear, with parallel margins (18-0)), J) *Ganapsis* sp. (arranged in one rows (11-0), present dorsally and ventrally (12-1), 3–5 SP *per* row (13-0), widely separated in a row (14-0), almost flat (15-0), with surface constantly plane (16-0), only reaching the distal margin of F_n_ (17-0), linear, with parallel margins (18-0)), K) *Ibalia rufipes* (arranged in three-four rows (11-2), present dorsally and ventrally (12-1), 6–8 SP *per* row (13-1), narrowly separated in a row (14-1), almost flat (15-0), with surface constantly plane (16-0), only reaching the distal margin of F_n_ (17-0), more or less sinuate (18-1)), L) *Oberthuerella* sp. (arranged in three-four rows (11-2), present dorsally and ventrally (12-1), >8 SP *per* row (13-2), closely spaced in a row (14-2), almost flat (15-0), with surface constantly plane (16-0), only reaching the distal margin of F_n_ (17-0), with parallel margins (18-0)).

The SP are the largest and the most conspicuous sensilla type on the antennae of all species (Fig. 4 and Fig. S2). In Cynipoidea, they are multiporous, elongate, plate-like sensilla with a large surface area (Fig. 4) (HAO reference: http://purl.obolibrary.org/obo/HAO_0000640). Length of SP ranged from 30–40 µm in Pediaspidini to 100–110 µm in *Iraella luteipes* (Thomson) (Aylacini I) (Table 2), with intermediate sizes spanned within all the other lineages. Even with the single genus *Andricus* (Cynipini), SP length varies greatly from 40–50 µm to 90–100 µm (Table 2). A similar observation arises when looking at SP width, though notably with a much more reduced variability, since it overall ranged from 2–4 µm (Anacharitinae) to 8–9 µm (Liopteridae) (Table 2).

Sensilla placoidea are rarely present on all flagellomeres (6 species spanning 4 tribes of Cynipidae and one subfamily of Figitidae) (Fig. S2), or starting from F_2_ (17 species across all families and most tribes), F_3_ (10 species across Cynipini and Aylacini I and II) or F_4_ (11 species across Figitidae and 4 tribes of Cynipidae, mainly Cynipini), so that all species have SP at least from F_5_ (characters from 7 to 10, Fig. 4 and Fig. S2). Sensilla placoidea are mostly arranged in one single row along the flagellomeres (character state 11-0, 37 species) with the remaining species having SP arranged in two (character state 11-1, 13 species), three or more rows (character state 11-2, 4 species). Basal cynipoids (Ibaliidae and Liopteridae) have 3 or more rows of SP; half of Figitidae have 1 row and half have 2 rows of SP. Within Cynipidae, Aylacini I and II and Cynipini mostly have 1 row of SP (all Cynipini have 1 row), while Diplolepidini, Qwaqwaini and half of gall-inquilines have 2 rows of SP, and Pediaspidini is the only cynipid tribe presenting ≥3 rows of SP. The Cynipini are particular in having SP, in most of cases (80%), only dorsally on the flagellum (character state 12-0: Fig. S2); only one species outside this tribe has the same pattern, *Synergus clandestinus* Weld (Synergini I). Sensilla placoidea can also vary in number within a single row (character 13: Fig. 4 and Fig. S2): 3–5 SP were visible in 20 species, mostly (18) within Cynipidae; 6–8 SP were detected in 29 species spanning all tribes of Cynipidae and including all Figitidae and Ibaliidae; of the 4 species presenting >8 SP per row, 3 were Cynipidae in three tribes and 1 was the liopterid. Considering together the number of SP rows (character 11), the dorsal or dorso-ventral presence of SP (character 12) and the number of SP per row (character 13) we can give a rough approximation of the overall number of SP in the F_n_. Thus, Liopteridae and Ibaliidae have by far the highest number of SP (4 SP rows × >8 SP per row both dorsally and ventrally). The figitids *Apocharips* sp. and *Ganaspis* sp. would have a very low number of SP (1 SP rows × 3–5 SP per row, both dorsally and ventrally), but the sexual form of three species of *Andricus* (Cynipini) (*A. burgundus* Giraud, *A. crispator* Tschek, *A. quercusradicis* (Fabricius)), having 1 SP row, SP only dorsally and 3–5 SP per row, may have the lowest SP number. *Hedickiana levantina* (Hedicke) (Aylacini I), with 3 SP rows and >8 SP per row both dorsally and ventrally, would be the species with more SP within Cynipidae, followed by the basal tribe Pediaspidini, with 3 SP rows and 6–8 SP per row both dorsally and ventrally.

The variation in the number of SP per row is associated with the relative spacing of SP in a row (character 14: Fig. 4 and Fig. S2). More than half of species (including most Cynipini and almost all Synergini (I+II)) have SP widely separated (> as width of SP); Aylacini I and Cynipini cover most of the species (11/16) presenting narrowly separated SP (< as width of SP), together with all the remaining cynipid tribes, most Figitidae and Ibaliidae; closely spaced, almost contiguous SP were found in only 3 species, including Liopteridae (Fig. S2).

Almost flat, only slightly or not rising on the segment SP (character state 15-0), were detected in all species but one, being the only exception *Acanthaegilips* sp. (Anacharitinae), which has ridge-like, clearly raising on the segment SP (character state 15-1) (Fig. 4 and Fig. S2).

Pairwise, almost all species (46) possess a SP with a surface always constantly smooth (character state 16-0), with only 5 species (all within Cynipidae) possessing a SP with at least some distinct longitudinal groove (character state 16-1) (Fig. 4 and Fig. S2). Sensilla placoidea can also vary depending on whether they at most only reach the distal margin of the flagellomere or if they more or less overlap the distal margin of the flagellomere (character 17: Fig. 4 and Fig. S2). Ibaliidae, Liopteridae, most Aylacini (I+II), Eschatocerini, Paraulacini and Pediaspidini do not have SP overlapping the margin, while Cynipini, Qwaqwaiini, Synergini (I+II) have clearly SP overlapping the margin. Figitidae presented a mixed situation, even within single subfamilies (Plectocynipinae).

Along the flagellomere, the SP can develop roughly linear (most Aylacini I+II, most Cynipini, most Synergini, most Figitidae and Liopteridae) (character state 18-0) or more or less sinuate (Ibaliidae, Eschatocerini, Paraulacini, Qwaqwaiini and Pediaspidini and the few remaining species from the other groups) (character state 18-1 (Fig. 4 and Fig. S2).

#### Sensilla coeloconica (SCo-A, SCo-B)

**Figure 5:**
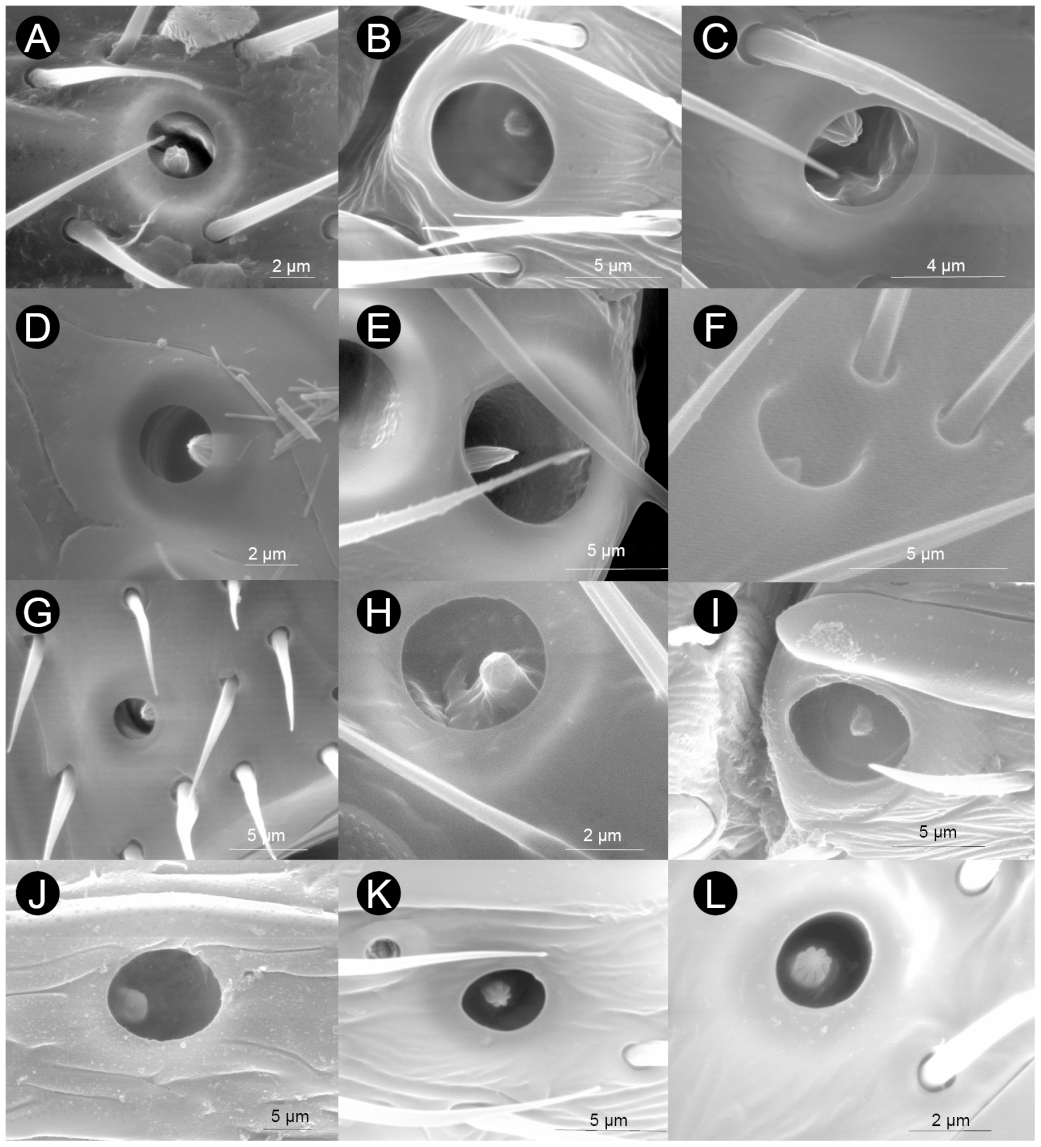
Examples of sensilla coeloconica type A (SCo-A) found in the flagellomere F_n_ of Cynipoidea. A) *Aulacidea tragopogonis*, B) *Andricus curvator* (sexual), C) *Periclistus brandtii*, D) *Neralsia* sp., E) *Pediaspis aceris* (asexual), F) *Timaspis phoenixopodos*, G) *Callaspidia notata*, H) *Ceroptres cerri*, I) *Dryocosmus kuriphilus* (asexual), J) *Eschatocerus acaciae*, K) *Iraella luteipes*, L) *Xestophanes potentillae*. Note the variability in the diameter of the SCo pit.

**Figure 6:**
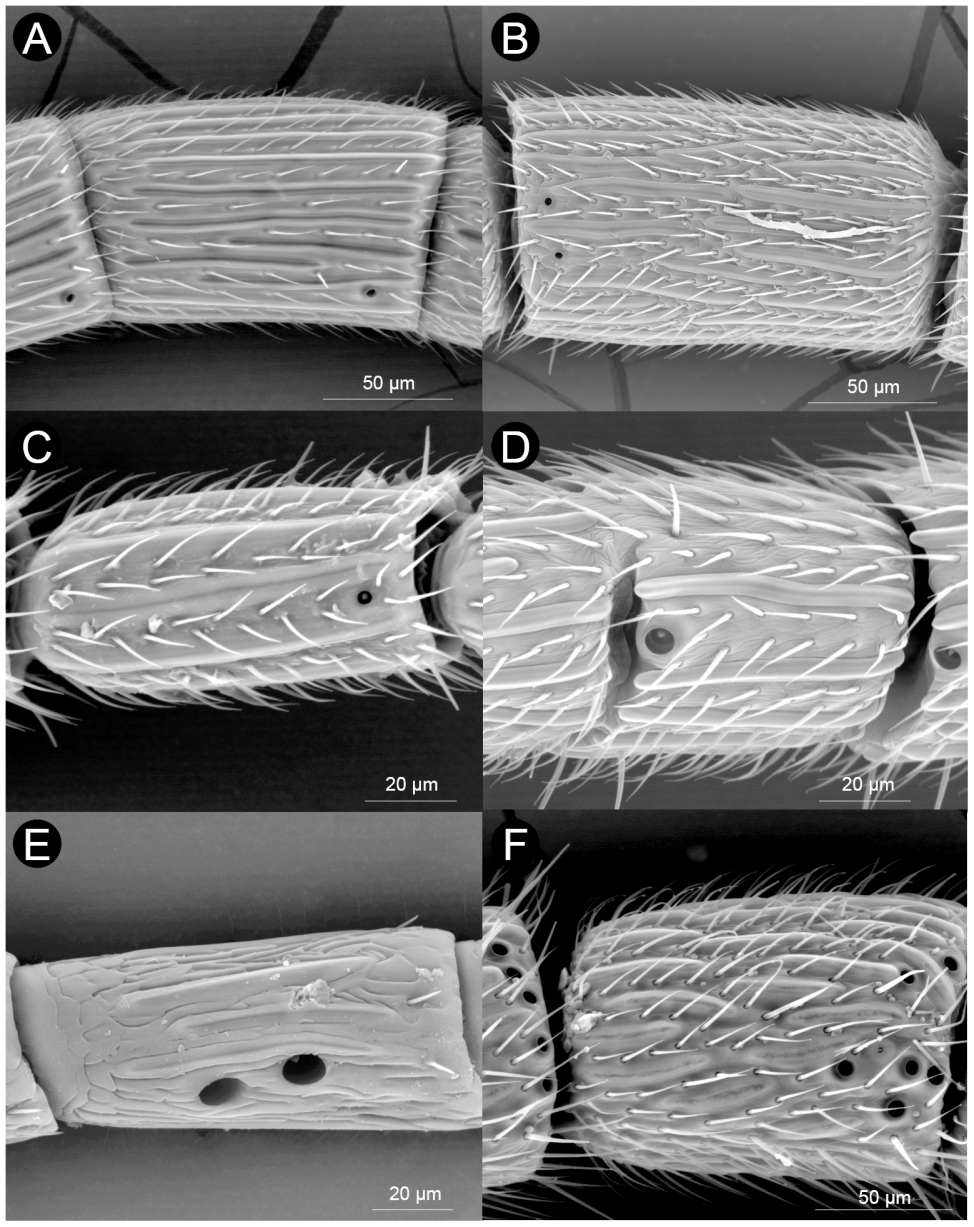
Variability in number, relative size and arrangement of sensilla coeloconica type A (SCo-A) in the flagellomere F_n_ of Cynipoidea. A) *Acanthaegilips* sp. (one *per* flagellomere (21-1), far from the distal margin (22-1), small (23-0)), B) *Hedickiana levantina* (≥3 *per* flagellomere (21-3), far from the distal margin (22-1), small (23-0)), C) *Aulacidea tragopogonis* (one *per* flagellomere (21-1), far from the distal margin (22-1), small (23-0)), D) *Andricus curvator* (sexual) (one *per* flagellomere (21-1), on or close the distal margin (22-0), large (23-2)), E) *Eschatocerus acaciae* (two *per* flagellomere (21-2), far from the distal margin (22-1), large (23-2)), F) *Pediaspis aceris* (asexual) (≥3 *per* flagellomere (21-3), on or close the distal margin (22-0), medium size (23-1)).

**Figure 7:**
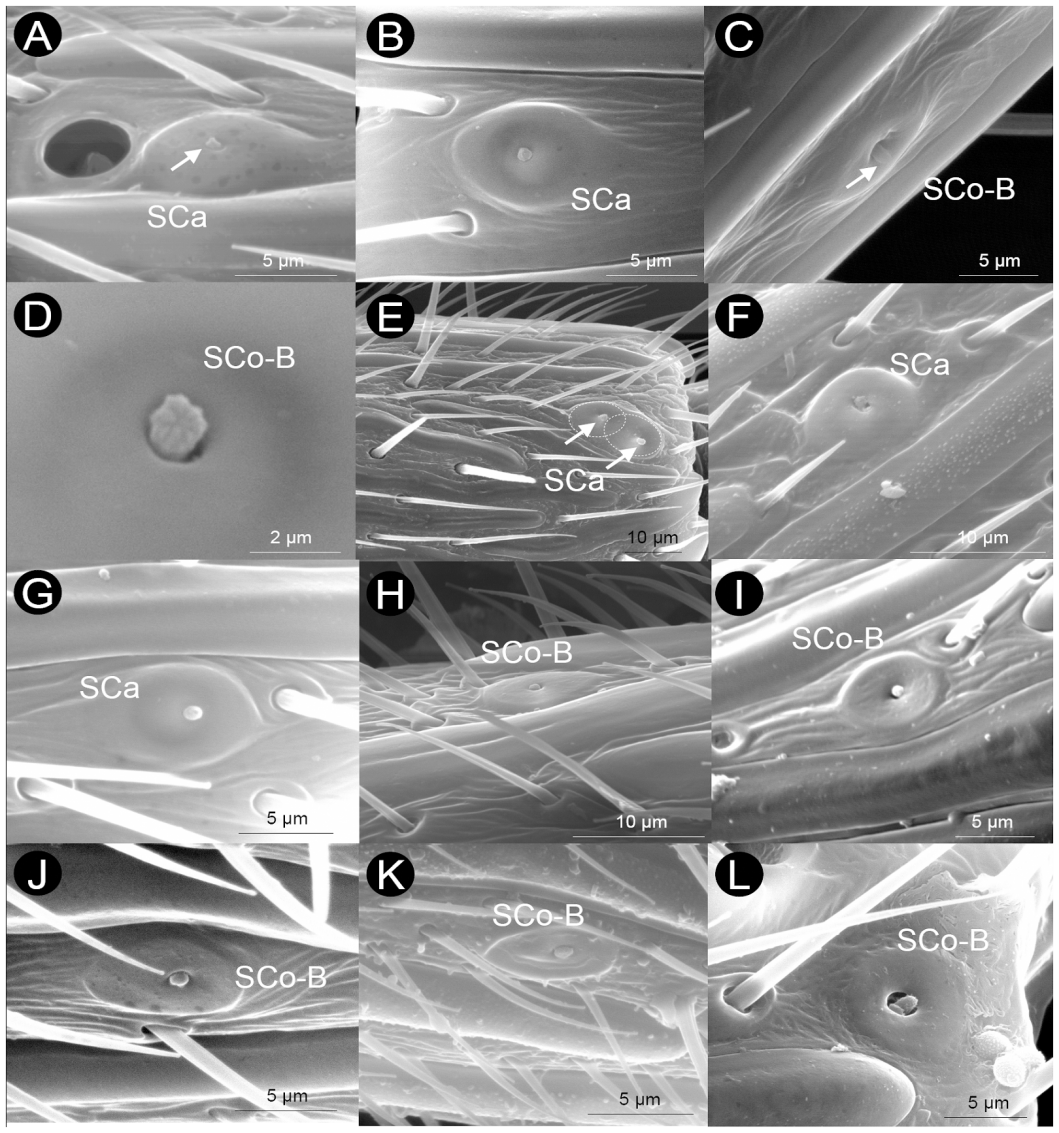
Examples of sensilla coeloconica type B (SCo-B) and sensilla campaniformia (SCa) found in the flagellomeres of Cynipoidea. A) *Andricus quercusilicis* (sexual) (arrow poiting at the peg of SCa), B) *Andricus burgundus* (sexual), C) *Diastrophus rubi* (arrow poiting at the peg of SCo-B), D) *Cecinothofagus gallaelenga*, E) *Qwaqwaia scolopiae*, F) *Hedickiana levantina*, G) *Andricus curvator* (sexual), H) *Isocolus lichtensteini*, I) *Andricus grossulariae* (sexual), J) *Andricus multiplicatus* (sexual), K) *Ceroptres cerri*, L) *Pediaspis aceris* (asexual). Note that these two types of sensilla are overall similar but in SCa the peg a bit smaller and is on the top of a doomed area, while in SCo-B a slightly larger peg visibly arises from a pit in a less doomed and even often depressed, concave area. Note also a rare case of a pair of SCa in E (arrows).

Sensilla coeloconica are recessed in deep pits. These are poreless sensilla composed of a cuticular peg standing on the antennal surface and possessing a “collar” of wrinkled cuticle surrounding the peg, which is set in a distinct cuticular depression (pit) (Figs. 5–6 and Fig. S3) (HAO reference: http://purl.obolibrary.org/obo/HAO_0002001). The peg is very bulbous, with the stalk of the peg giving rise to finger-like projections joining at the tip. We found two types of SCo, SCo-A (HAO reference: http://purl.obolibrary.org/obo/HAO_0002304) and SCo-B (HAO reference: http://purl.obolibrary.org/obo/HAO_0002305). The two types differ in two main aspects. First, SCo-A are much larger than SCo-B (Figs. 5–7). Second, the peg/pit diameter ratio is roughly 1/3-1/5 in SCo-A and essentially invariably 1∶1 in SCo-B.

Basal families lack SCo-A (Liopteridae) or have SCo-A only in F_A_ (Ibaliidae). In Charipinae, Paraulacini and two species of Synergini I this sensilla type seems also to be absent. Sensilla coeloconica type A are located ventrally, sometimes ventro-laterally (Fig. 6 and Fig. S3). They typically start in the middle part of flagellum (F_5_-F_8_ to F_n_) (character state 19-0: Fig. S3, 38 species), while sometimes they start in the proximal part of flagellum (F_2_-F_4_ to F_n_) (character state 19-1). Sensilla coeloconica type A are normally present up to F_A_ (42 species) (character state 20-0), while in some cases it was absent in F_A_ (character state 20-1).

Sensilla coeloconica type A are present in relatively low numbers in each flagellomere, though certain variability appears (character 21: Fig. 6 and Fig. S3). In most cases one SCo-A is present (36 species), though on rare occasions, SCo-A were found in pairs (6 species) or clusters of three or more on a single flagellomere. Pediaspidini were by far those with the highest number of SCo-A in a flagellomere (up to 7, Fig. 6). Most Figitidae, Aylacini I+II, Diplolepidini and Eschatocerini have SCo-A far from the flagellomere's distal margin (character state 22-0: Fig. 6 and Fig. S3), while Cynipini, Pediaspidini and Synergini I+II have SCo on or close to the distal margin (character state 22-1). The pit of SCo-A has a diameter mostly ranging from 2.5 µm to 5 µm (42 species) (Table 2). Only four species has a SCo-A pit wider than 5 µm (Pediaspidini, Eschatocerini and two Cynipini), with *Eschatocerus acaciae* Mayr having by far the largest SCo-A pit (11 µm) (Table 2, Fig. 5). The peg of SCo-A was much less variable in size, having a diameter of 1 µm in all species except *Dryocosmus kuriphilus* Yasumatsu (asexual) (1.5 µm) and *E. acaciae* (2 µm). When pit size was related with F_n_ width (character 23: Fig. 6 and Fig. S3), it appears that Figitidae, most Aylacini (I+II) and Diplolepidini tend to have smaller SCo-A (compared to F_n_ width) than Cynipini, Eschatocerini and Pediaspidini. When considering together the maximum number of SCo-A in a flagellomere (1, 2 or 3 (3 indicating ≥3) and the size of SCo-A relative to F_n_ (rank: 1 to 3), we obtained a picture in which wood-gallers seem to have overall a greater portion of the F_n_ covered by SCo-A compared with the other lineages. Eschatocerini (due to the greater pit size) and Pediaspidini (due to their greater number of SCo-A) were the groups with the highest values.

Sensilla coeloconica type B occurred in 38 taxa and have similar morphology across them (Fig. 7, Table 2). Sensilla coeloconica type B are apparently absent in three gall-inquilines, in Diplolepidini, in five Figitidae, in two Cynipini, in two Aylacini I, in Liopteridae and in Ibaliidae. They are small sensilla with a peg of 1.5 µm in diameter roughly occupying the whole pit (thus very differently than SCo-A). The pit is located in a flattened or even depressed area of the flagellomere, about 5–11 µm in diameter (but these values are much approximated since it is difficult find precise bounds of such area). No more than one sensillum per flagellomere was observed.

#### Sensilla campaniformia (SCa)

Sensilla campaniformia are characterized by a button-like knob about 1 µm in diameter with a small irregular surface emerging from an opening in the centre of a domed, smooth, circular cuticular disk (Fig. 7, Table 2) (HAO reference: http://purl.obolibrary.org/obo/HAO_0001973). This dome is about 5–10 µm in diameter, but, as in case of SCo-B, it is very difficult to find precise bounds of such area, which gently grade progressively up to the same level with the antennal surface (Fig. 7). Sensilla campaniformia are quite rare along the antenna, typically with a maximum of one sensillum per flagellomere (2 fused SCa were extremely rarely observed, as in *Qwaqwaia scolopiae*, Fig. 7), and often close to the SCo-A (Fig. 7). Such a sensilla type was found in most species (43), apparently lacking in three gall-inquilines, in Diplolepidini, in one Cynipini, in four Figitidae and in Liopteridae (Table 1). Their morphology resembles the SCo-B, but in the latter case, the slightly larger peg visibly protrudes from a pit in a less domed, often depressed, concave area (Fig. 7).

#### Sensilla basiconica (SB)

**Figure 8:**
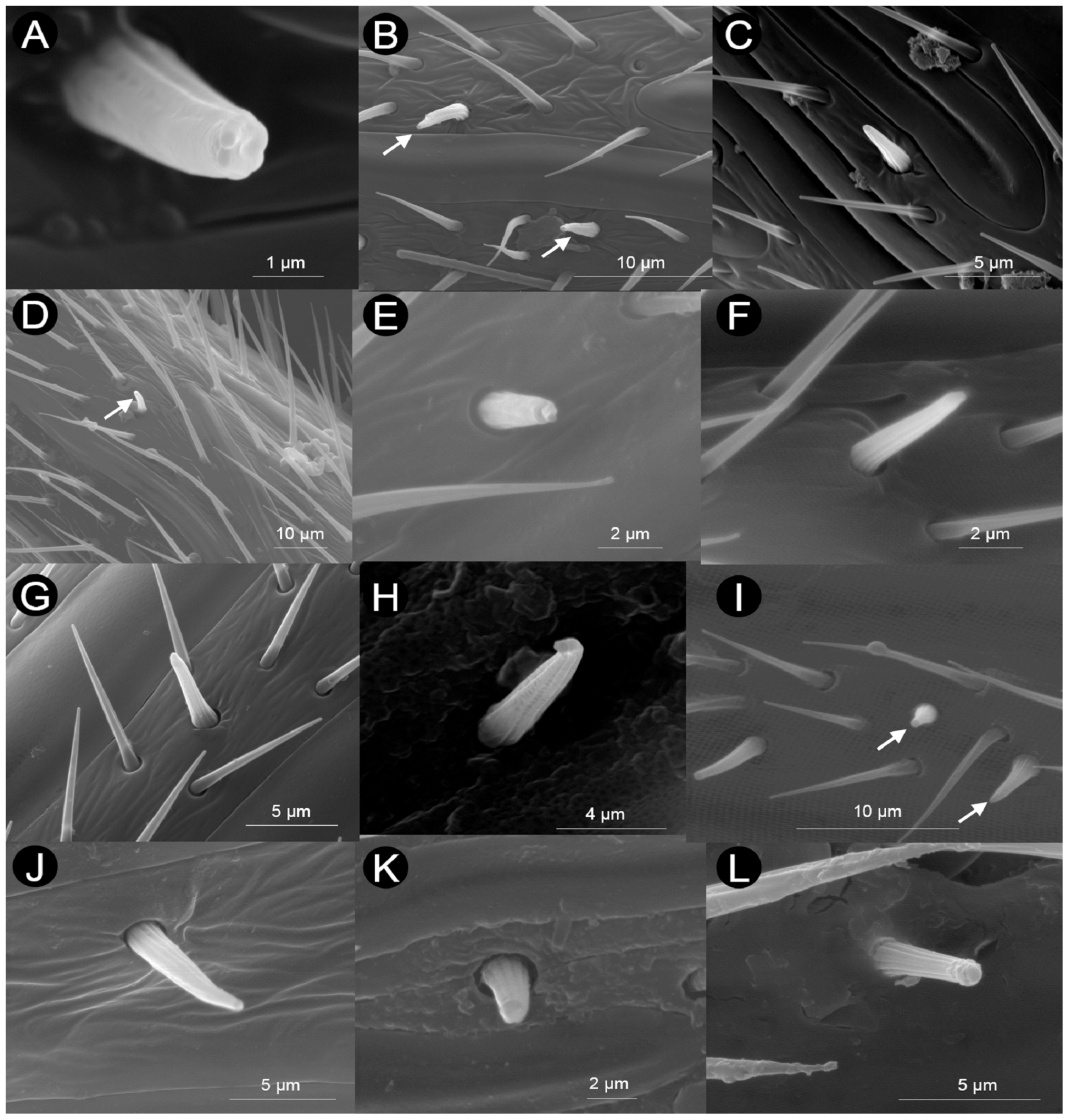
Examples of sensilla basiconica (SB) found in the flagellomeres of Cynipoidea. A) *Andricus corarius* (asexual), B) *Periclistus brandtii*, C) *Roophilus loewi*, D) *Trigonaspis sinaspis* (sexual), E) *Andricus multiplicatus* (sexual), F) *Synergus physocerus*, G) *Synergus clandestinus*, H) *Aylax papaveris*, I) *Callaspidia notata*, J) *Diastrophus rubi*, K) *Qwaqwaia scolopiae*, L) *Oberthuerella* sp. Note one small and one large SB in I.

Sensilla basiconica were detected in most species (48), apparently lacking in Plectocynipinae, Eucoilinae, Eschatocerini and one Aylacini I (*Liposthenes kerneri* (Wachtl)) (Table 1). These sensilla are hair-like, characterized by a grooved surface, and project almost perpendicularly with respect to the axis of the antenna (Fig. 8) (HAO reference: http://purl.obolibrary.org/obo/HAO_0002300). The pegs of SB arise from a shallow socket and they are generally not curved, though sometimes they are curved at their distal, tapered blunt and pored apex (Fig. 8). Sensilla basiconica can be easily differentiated under low magnification from sensilla trichoidea (see below), based on the relatively greater width at the base, greater overall thickness, and, at least compared with three types of sensilla trichoidea (ST-A, B, C, see below), on their relatively shorter length. The peg length ranged in most cases from 3 to 4 µm (Table 2); however, in about half of the species (23), we observed some very small SB, about 1.5 µm in length, together with others of the typical length (Table 2, Fig. 8).

#### Sensilla trichoidea (ST-A, ST-B, ST-C, ST-D, ST-E)

**Figure 9:**
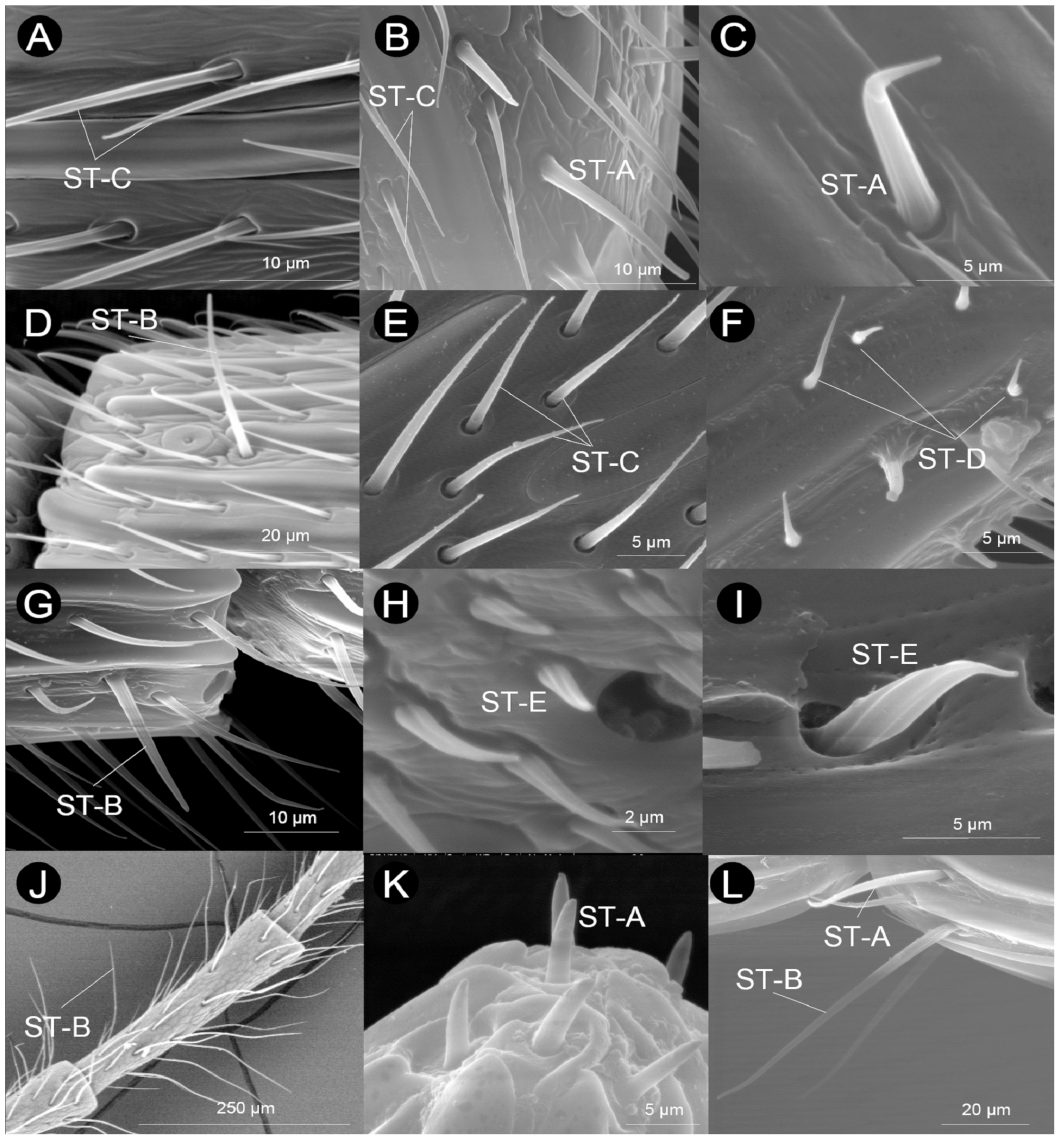
Examples of sensilla trichoidea (ST-A, ST-B, ST-C, ST-D, ST-E) found in the flagellomeres of Cynipoidea. A) *Andricus curvator* (sexual), B) *Andricus coriarius* (asexual), C) *Andricus grossulariae* (asexual), D) *Andricus grossulariae* (asexual), E) *Pediaspis aceris* (asexual), F) *Aulacidea tragopogonis*, G) *Aulacidea papaveris*, H) *Synergus hayneanus*, I) *Oberthuerella s*p., J) *Cynips quercusfolii* (asexual), K) *Eschatocerus acaciae*, L) *Neralsia* sp.

We found five different types of hair-like structures, which we overall named sensilla trichoidea (Fig. 9) (HAO reference: http://purl.obolibrary.org/obo/HAO_0002299). Overall, sensilla trichoidea were abundant on F_n_ (and in general on the whole flagellum) both ventrally and dorsally.

Sensilla trichoidea type A were widespread in our sample, occurring in all but one species (Table 1). This sensillar type consists in moderately long (from 5–6 µm to 7–15 µm) hair-like structures generally not perpendicular to the antennal axis (Fig. 9, Table 2). Their surface is finely grooved. Sensilla trichoidea type A in Eschatocerini are unique in being thicker than in the other species and being arranged in groups of 3–5 at the apex of F_A_ (Fig. 9).

Sensilla trichoidea type B were similar in their general shape to ST-A, but they are visibly longer (they are the longest sensilla trichoidea, ranging from about 10 to 40 µm in length, Table 2) and they are almost perpendicular to the antennal axis (Fig. 9). They also differ from ST-A and from the other sensilla trichoidea because of their typical arrangement on a flagellomere (mostly a pair is present in opposite sides close to the distal margin of the flagellomere, Fig. 9). Sensilla trichoidea type B were common, having been observed in 46 species (Table 1), notably lacking in basal families (Ibaliidae and Liopteridae). Despite the great variability in length within cynipid lineages, it seems that there is a certain tendency for Aylacini I+II and Synergini I+II to have shorter ST-B (up to 20 µm) than wood-gallers (Cynipini, Qwaqwaiini, Pediaspidini) (>20 µm) (Table 2).

Sensilla trichoidea type C were variably long sensilla (from 4–5 µm to about 40 µm), most often around 5–15 µm long (Fig. 9, Table 3); they were very widespread and occurred in all species, and they are those with highest density on the antennae. They are characterized by their strong inclination, almost laying on the antennal surface, and by their reduced thickness compared with ST-A and ST-B (Fig. 9). Very long or very short ST-C were found in closely related taxa. For example, among Figitidae, *Neralsia* sp. had 18–42 µm long ST-C while in *Acanthaegilips* sp. ST-C were not longer than 7 µm (Table 3). Within Aylacini I, *Aulacidea* spp. had 4–5 µm long ST-C while *Aylax papaveris* (Perris) had 10–17 µm long ST-C (Table 3).

**Table 3:** Measurements (in µm) taken on the antennae and sensilla of the studied species.

Taxon	F_n_ length	F_n_ width	SP length	SP width	SCo-A Pit	SCa Disc	SB	ST-A	ST-B	ST-C	ST-D	ST-E	LDS	LVS
*Acanthaegilips* sp.	115	90	50–100	2–4	3	–	3–4	7–11	–	5–7	–	–	–	–
*Andricus burgundus* (S)	56	55	50–60	4–5	5	8	1.5–4	6–12	14–17	8–13	–	–	–	–
*Andricus coriarius* (A)	95	80	90–100	5–6	5	8	3–4	7–12	21–26	10–12	–	–	–	–
*Andricus crispator* (S)	67	54	50–60	4–5	4	7	3–4	8–12	14–20	10–13	–	–	–	–
*Andricus curvator* (S)	57	56	60–80	3–5	6	7	3–4	8–14	17–20	11–13	–	–	–	–
*Andricus grossulariae* (A)	97	81	80–90	5–6	5	7	3–4	7–10	22–32	9–13	–	–	–	–
*Andricus grossulariae* (S)	73	63	50–70	5–7	3	6	3–4	8–14	10–15	10–11	–	–	–	–
*Andricus multiplicatus* (S)	69	68	70–80	5–6	4	8	1.5–4	7–12	14–20	8–14	–	–	–	–
*Andricus pictus* (A)	96	74	90–100	5–6	5	7	1.5–4	7–12	22–27	11–12	–	–	–	–
*Andricus quercusradicis* (A)	110	95	80–100	6–8	5	7	3–4	6–12	21–25	10–13	–	–	–	–
*Andricus quercusradicis* (S)	93	64	70–80	5–6	4	6	3–4	9–13	18–24	9–14	–	–	–	–
*Andricus quercusramuli* (S)	51	48	40–50	4–5	4	7	1.5–4	8–11	11–16	11–13	–	–	–	–
*Apocharips* sp.	61	29	50–60	4–5	–	6	3–4	9–11	8–12	5–11	–	–	–	–
*Araucocynips queulensis*	65	53	40–50	4–5	3.5	–	–	9–11	11–15	7–13	1–1.5	_	6–7	_
*Aulacidea freesei*	62	50	70–80	4–6	2.5	5	1.5–4	6–13	12–20	4–5	1–2	–	–	–
*Aulacidea tragopogonis*	86	45	70–90	6–7	3.5	6	1.5–4	7–15	18–24	4–5	2–4	–	–	–
*Aylax papaveris*	112	57	90–110	6–7	3.5	7	3–4	6–12	16–20	10–17	–	–	–	–
*Callaspidia notata*	140	88	70–100	4–5	2.5	9	1.5–4	6–13	–	4–8	–	3–6	–	–
*Cecinothofagus gallaelenga*	81	79	40–70	6–7	–	7	3–4	7–12	20–23	6–11	2–4	–	–	13–15
*Ceroptres cerri*	56	46	50–60	4–5	4	6	3–4	8–11	–	8–16	–	–	–	–
*Cynips quercusfolii* (A)	75	80	70–80	6–8	3	8	3–4	8–10	17–23	7–16	–	–	–	–
*Diastrophus rubi*	96	66	80–90	4–5	4	6	3–4	8–14	20–21	18–26	–	–	–	–
*Diplolepis rosae*	105	68	60–80	4–6	3.5	–	1.5–4	7–12	23–27	15–21	–	–	–	–
*Dryocosmus kuriphilus* (A)	69	51	70–80	5–6	7	7	3–4	8–13	17–20	17–23	–	–	–	–
*Eschatocerus acaciae*	83	49	40–70	5–8	11	10	–	5–6	–	5–8	–	–	–	–
*Ganaspis* sp.	80	38	70–80	4–5	2.5	–	–	–	25–32	25–29	–	–	–	–
*Hedickiana levantina*	136	83	50–70	4–6	3	8	1.5–4	7–11	–	7–11	–	–	–	–
*Ibalia rufipes*	228	153	50–70	5–7	3.5	11	3–4	6–11	–	5–9	–	–	–	–
*Iraella luteipes*	118	47	100–110	7–8	4	8	3–4	9–11	20–25	11–16	–	–	–	–
*Isocolus lichtensteini*	139	71	60–100	5–8	3.5	8	1.5–4	8–12	13–17	6–9	2–3	–	–	–
*Liposthenes kerneri*	69	46	70–80	4–5	4	5	–	8–11	16–20	8–12	2–3	–	–	–
*Neralsia* sp.	114	82	80–100	4–6	3	5	1.5–4	9–13	33–40	18–42	4–7	–	–	–
*Oberthuerella sp.*	338	323	70–100	8–9	–	–	1.5–4	8–12	–	7–12	–	10–14	–	–
*Panteliella fedtschenkoi*	70	42	50–60	6–7	2.5	8	3–4	6–12	12–13	10–14	–	–	–	–
*Parnips nigripes*	138	78	50–70	5–6	4	6	3–4	9–11	18–20	14–18	–	–	–	–
*Pediaspis aceris* (A)	95	92	30–40	6–7	5.5	9	3–4	7–14	25–34	10–23	–	–	–	–
*Periclistus brandtii*	130	62	60–100	4–5	3.5	6	1.5–4	7–11	17–20	12–16	3–4	–	–	–
*Phanacis centaureae*	72	43	60–70	7–8	3	7	3–4	7–9	13–15	6–9	–	5–7	–	–
*Plagiotrochus quercusilicis* (S)	58	37	60–70	5–6	4	7	3–4	8–11	18–24	7–11	–	–	–	–
*Plectocynips pilosus*	80	73	70–90	5–6	4	–	–	9–11	20–25	10–16	–	–	10–12	–
*Pseudoneuroterus macropterus* (A)	72	59	50–60	4–5	3.5	7	3–4	9–12	17–25	5–11	3–4	–	–	–
*Qwaqwaia scolopiae*	78	77	50–70	4–5	–	9	1.5–4	8–11	26–32	10–12	–	–	–	–
*Rhoophilus loewi*	81	44	50–70	4–6	–	8	1.5–4	6–10	13–15	7–11	–	–	–	–
*Saphonecrus lusitanicus*	62	45	50–60	3–4	2.5	–	1.5–4	6–9	10–17	6–9	–	–	–	–
*Synergus clandestinus*	74	56	60–70	5–7	–	–	1.5–4	8–11	13–14	7–10	–	–	–	–
*Synergus hayneanus*	102	47	50–70	5–6	3.5	7	1.5–4	8–11	14–15	5–6	2–3	3–5	–	–
*Synergus physocerus*	71	38	50–70	4–5	3	8	1.5–4	7–12	13–14	6–10	–	–	–	–
*Synergus umbraculus*	111	53	70–80	4–5	–	–	1.5–4	8–11	14–16	7–12	–	–	–	–
*Synophrus politus*	115	83	50–80	4–5	4	8	1.5–4	6–12	14–15	7–10	–	–	–	–
*Timaspis phoenixopodos*	81	45	50–60	6–7	3.5	7	1.5–4	7–14	20–25	10–16	2–3	–	–	–
*Trigonaspis mendesi* (A)	62	64	60–70	6–9	3	8	3–4	8–11	19–25	23–32	4–5	–	–	–
*Trigonaspis synaspis* (S)	162	75	70–90	5–7	5	7	1.5–4	8–14	25–30	20–23	–	–	–	–
*Xestophanes potentillae*	97	62	60–70	5–6	3	8	3–4	8–13	15–16	8–16	–	4–5	–	–

The measures of the peg of the SCa (always 1 µm) and the pit and peg of SCo-B (always 1.5 µm) are not reported. The diameter of SCo-A peg is not reported since it is always 1 µm except in two species (data presented in the Results) “–” appears if that sensillar type is absent in that species. When more than one sensilla were measured for a given sensillar type and taxon, the minimum-maximum range is reported.

Sensilla trichoidea type D were not very abundant in our sample, having been observed in only 12 species (7 herb-gallers, 4 gall-inquilines and one non-gall parasitoid) (Table 1). They are short hair-like sensilla ranging from 1–2 µm to 4–7 µm in length (Fig. 9, Table 2). They are typically bulbous at the base (Fig. 9).

Sensilla trichoidea type E were the rarest sensilla trichoidea on cynipoid antennae, having been detected in only five species (Liopteridae, Aspicerinae and three Cynipidae) (Table 1). These sensilla are easily recognized by their twisted/spiral grooves, their great thickness and their curved apex (Fig. 9). They ranged from 3 to 7 µm in length in all cases except in *Oberthuerella* sp. (Liopteridae), which has 10–14 µm-long ST-E (Table 2).

Considering sensilla trichoidea as a whole, we found that most species (42) have sensilla trichoidea of similar length in F_1_ and F_A_ (character state 33-0: Table 1 and Fig. S4). However, six Cynipini, *Acanthaegilips* sp. (Figitidae), Paraulacini, Pediaspidini and Liopteridae have F_1_ with slightly different sensilla trichoidea than F_A_ (character state 33-1). The cynipine *Cynips quercusfolii* L. (asexual) was the only studied species having a very strong difference in sensilla trichoidea between F_1_ and F_A_, since it posses extremely long sensilla trichoidea (100–130 µm) in F_1_ (character state 33-2). The number of sensilla trichoidea (as a whole) on F_n_, measured in a row along its length, also varied among species (character 34: Table 1 and Fig. S4). Eschatocerini and two figitids have very few (1–2) sensilla trichoidea; 30 species, mostly Cynipini and Aylacini I+II (22 species), have 4–9 sensilla trichoidea; 17 species, mostly gall-inquilines (nine species), have 10–15; and three species, all non-gall parasitoids, have very dense and abundant sensilla trichoidea (>15) (Fig. S4). It should be noted that, despite the fact that we could not count sensilla trichoidea of each type, most of the variation in density is likely to be due to variation in ST-C density, since they were by far the most abundant sensillar type on the antennae.

#### Large disc sensilla (SLD)

**Figure 10:**
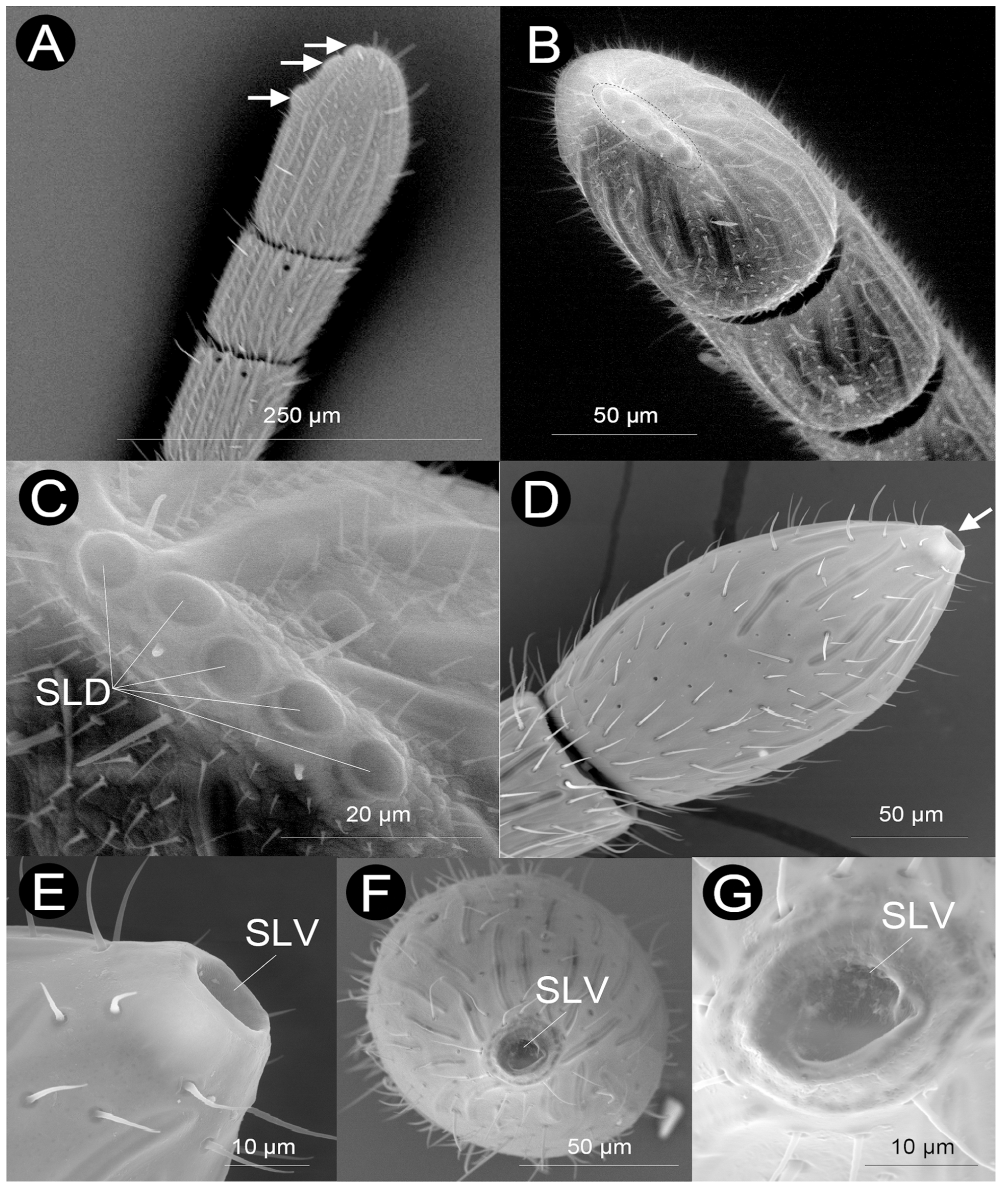
Unique sensilla types found on the apex of the antennae of Paraulacini (Cynipidae) and Plectocynipinae (Figitidae). A) last three flagellomeres of *Plectocynips pilosus* (Plectocynipinae), with arrows pointing the three-disc Large Disc Sensilla (SLD), B) last three flagellomeres of *Araucocynips queulensis* (Plectocynipinae), with arrows pointing the five-disc Large Disc Sensilla (SLD), C) Detail of the SLD in *A. queulensis* (Plectocynipinae), D) apical clava of *Cecinothofagus gallaelenga* (Paraulacini), with arrow poiting the Large Volcano Sensilla (SLV), E) lateral view of the SLV in *C. gallaelenga* (Paraulacini), F) frontal view of the SLV in *C. gallaelenga* (Paraulacini), G) detail of the cone entrance of the SLV in *C. gallaelenga* (Paraulacini).

This type of sensilla was exclusively found in Plectocynipinae, which include at least one gall-inquiline genus (*Plectocynipis*), and only at the ventral side of F_A_, near to the apex (Fig. 10). As far as we know this type of sensilla had not been described before and we here name them as “large disc sensilla” (HAO reference: http://purl.obolibrary.org/obo/HAO_0002303). Large disc sensilla are composed of a number of large, roughly oval-circular, discs, each 6–7 (*Araucocynips queulensis* (Buffington & Nieves-Aldrey)) or 10–12 (*Plectocynips pilosus* (Ros-Farre)) µm in diameter. Three discs were counted in *P. pilosus* and five in *A. queulensis* (Fig. 10). The discs of the row are located on a single plate rising from the antennal cuticle. The discs do not posses a peg as occurs in SCa and are more oval in shape than SCa.

#### Large volcano sensilla (SLV)

This type of sensilla was exclusively found in the gall-inquiline Paraulacini (*Cecinothofagus gallaelenga* Nieves-Aldrey & Liljeblad), and only at the apex of F_A_ (Fig. 10). As in the case of SLD, we could not find any description of this type of sensilla in the literature, so here we name them “large volcano sensilla” (HAO reference: http://purl.obolibrary.org/obo/HAO_0002302). These sensilla have a “volcano” shape, i.e. a large conical structure of 13–15 µm of diameter. No pegs or other protruding structures were found arising from this large cone, though we cannot exclude that some additional structures are deeply recessed within the cone.

## Discussion

The present study is the first to characterize the antennal sensillar equipment and the antennal morphology across all the tribes of Cynipidae and concerning many lineages of Cynipoidea as a whole. This represents a great advance, since, as far as we know, the only studies on antennal sensory structures of Cynipoidea concerned *Tribliographa rapae* Westwood (Figitidae), two species of *Aganaspis* (*A. daci* (Weld) and *A. pelleranoi* (Brèthes)) (Figitidae), and the cynipid *D. kuriphilus* [48]–[50]. On the whole, our results show that the sensillar equipment on the antennae of Cynipoidea have some similarities with that described for these previously studied species, as well as some similarities with that of other species of parasitoids from different families of Hymenoptera. Comparisons, especially with groups phylogenetically closer to Cynipoidea (i.e, those in the Proctotrupomorpha, i.e. Chalcidoidea, Platygastroidea, Proctotrupoidea, Diaprioidea and Mymarommatoidea, plus the Ichneumonoidea, the sister group to Proctotrupomorpha [68]), are presented below. In particular for Cynipidae, we also discussed the possible evolutionary paths of some characters, taking into account the most recent phylogeny (Fig. 1) and the possible links between characters' variability and certain life-history traits.

Although the nomenclature used to describe the different types of sensilla is not uniform across literature on Hymenoptera, we propose potential homologies of certain types of sensilla among hymenopteran lineages, based on similarities of their external morphology. However, such proposed homologies should be confirmed in future by histological studies.

Cynipoidea females possess, with very few exceptions, a filiform antenna of 10–13 flagellomeres. One exception is the figitid subfamily Pycnostigminae, whose species possess antennae of more than 15 flagellomeres [69]. Within Proctotrupomorpha, other antennal morphologies can be found. For example, geniculate and clavate antennae (i.e. bent or hinged sharply, almost like a knee or elbow joint, with an apical clava or club) can be found in the majority of Chalcidoidea and in Platygastroidea [22], [70]–[73], while in Ichneumonoidea, the antennae are more often filiform (often of the moniliform sub-type) [74], [75]. It seems, thus, that Cynipoidea have overall antennae with a general shape more similar to Ichneumonoidea than the rest of Proctotrupomorpha. The number of flagellomeres within Proctotrupomorpha is also variable, with an apparent reduction of the number of flagellomeres in Chalcidoidea and Mymarommatoidea (often less than 10) compared with Cynipoidea (10–13) (but see Pycnostigminae), Platygastroidea and Proctotrupoidea (12–15) [76]–[79]. Our data furthermore suggest that, during the evolution of Cynipoidea, a certain shift towards longer F_1_ (compared with F_2_ length) occurred in Cynipidae.

The total number of sensilla types observed in female Cynipoidea ranged from 4 to 10 depending on species, with 12 different types described in the superfamily as a whole. In the other Proctotrupomorpha studied to date, the number of sensilla types ranged from 4 to 14 per species [11], [22], [70]–[73], [80], and 4–11 types per species were described in Ichneumonoidea [26], [74], [75]. A comparison of the morphology of these sensillar types among lineages is presented below, together with hypotheses on their function as suggested by histological studies performed on some species.

The sensilla placoidea (SP) are very common among the apocritan Hymenoptera, being typically fewer, larger, and more elongated in the Proctotrupomorpha and Ichneumonoidea, and very abundant, smaller and more circular in the Aculeata, particularly in the Apoidea (bees and apoid wasps) [33], [51]. According to our study and the other few studied species in the past [48]–[51], in Cynipoidea, SP are invariably elongate, plate-like and multi-porous sensory organs distributed on all or most flagellar segments and they are by far the largest of all other sensillar types. In the HAO portal, such sensillar type responds to the name of longitudinal sensillum, with other terms such as sensilla placoidea and multiporous plate sensillum treated as synonyms [56]. We suggest using sensilla placoidea as the main term for Hymenoptera as a whole, since not all sensilla of this type are longitudinal and elongated (e.g. in the Apoidea, see above). Contrary to what was previously reported [81], SP are also present in *Oberthuerella* (Liopteridae). Another liopterid genus, *Liopteron*, was previously proved to possess SP [51]. Concerning SP shape, some differences appear between Cynipoidea and other parasitic hymenopterans. For example, in some Chalcidoidea, SP have a different morphology than in Cynipoidea, being ridge-like with apices free and extending beyond apex of segment [82]. In other Chalcidoidea, however, SP are parallel to the surface and not attached to the antennal surface except proximally [83], similar to putative SP found in Platygastroidea (see below). On the contrary, SP are slightly flatter (though often still a bit more elevated than in Cynipoidea) in Ichneumonoidea, in some cases with longitudinal grooves similarly to those observed by us in some cynipids [26], [74], [75].

Among the other Proctotrupomorpha, SP similar to those here described for Cynipoidea occur only in Pelecinidae (Proctotrupoidea) [51]. In Platygastroidea, SP of the above-described shape seem to be absent. Instead, one type of multiporous sensilla, the papillary sensilla [73], could be homologous to SP, as previously also suggested by Bin [84], Barlin & Vinson [85], Basibuyuk & Quicke [51] and, Zacharuck [86] which called such sensilla as “plates” or “multiporous plates”. The very typical shape of papillary sensilla in Platygastroidea include their relatively small size (resembling the relative size of SP found in Aculeata, e.g. [33]) and their protruding, flattened and grooved surface within a nearly ellipsoid pit. However, according to Cave and Gaylor [80], the papillary sensilla should be considered as basiconic, and not as placoid, sensilla. As a matter of fact, they seem to be associated with tasting, being also named “multiporous gustatory sensilla” [21]. Another sensillar type very peculiar in Platygastroidea is the “sensillum trichodeum curvatum” or “horn- and sickle-like sensilla” [73], [80]. These are large, multi-porous, sharply bent anteriorly just above the base and are acutely pointed at the tip, and resemble the SP in Chalcidoidea and in *Acanthaegilips* sp., with the exception that these sensilla are not embedded in the antenna as in the SP. Such sensilla, and not the papillary sensilla, could be the homolog of SP in Platygastroidea, a hypothesis partially supported also by the fact that neither in the other Proctotrupomorpha nor in Ichneumonoidea these “horn- and sickle-like sensilla” are present. Furthermore, as for SP, their role seems to be related to sense of smell, due to their thin wall and the presence of many tubular pores [86]. Thus, overall, SP morphology strongly differs between Cynipoidea and Platygastroidea, which are closely related within the Proctotrupomorpha [67], and Cynipoidea share more similarities with Ichneumonoidea than with the phylogenetically closer Chalcidoidea.

Interestingly, Cynipoidea are apparently unique within Proctotrupomorpha + Ichneumonoidea in having some species (about 1/3 of the studied taxa) with more than one row of SP per flagellomere (though if we consider the “horn- and sickle-like sensilla” homologous to SP, Platygastroidea could also have this character). Also the number of SP per row seems to be higher in most Cynipoidea (>5 in 33 of the studied species) than in the other Proctotrupomorpha and in Ichneumonoidea (apparently no more than 4) [22], [26], [71]–[75], [80], which results in Cynipoidea having often SP narrowly or closely spaced in a row.

The main differences of SP among the studied Cynipoidea taxa concern their arrangement and relative size. Although in general the variability of such characters is great even within lineages, we can preliminarily propose at least one evolutionary trend in Cynipidae. In fact, it seems that there is a tendency during cynipid evolution to decrease the number of SP rows per flagellomere (character 11): from the basal clade Pediaspidini + Diplolepidini (state 1/2) there is a shift to the state 0/1 in all the remaining cynipid tribes except Cynipini (the most derived tribe) which only present the state 0. This trend is supported also by the fact that other cases of state 2 occurred only in basal Cynipoidea (Fig. 1 and Table 2). On the other hand, the number of SP per row is extremely variable within lineages and no evolutionary scenario can be pictured (Fig. 1 and Table 2). This is even apparent within species: for example, in the cynipine *A. quercusradicis*, the sexual form has 3–5 SP/row (state 0) and the asexual form has 6–8 SP/row (state 1).

The function of SP is assumed to be olfactory because they posses a multiple cuticular pore system [51], and electro-physiological research showed that SP in parasitoids are effectively olfactory receptors which responded in a dose-dependent manner to plant volatiles [23]. In Cynipoidea, SP are likely to be involved in host (Ibaliidae, Liopteridae, and most Figitidae) gall-host (gall-inquilines or gall-parasitoids) or plant-host searching and/or finding (gall-inducers), especially in the detection of long-range cues.

A coeloconic sensillum is defined, in the HAO portal, as an aporous sensillum that is peg-shaped and is located in a depression [56], thus agreeing with our overall definition. We suggest including the term sensilla coeloconica type A within the HAO portal to indicate sensilla coeloconica with a large pit with a comparatively small protruding peg, and the term sensilla coeloconica type B to indicate sensilla coeloconica with a small pit with a protruding peg occupying the whole pit; either one or both types of SCo are found in many hymenopteran groups (see below).

Sensilla coeloconica type A (SCo-A) are not very abundant on the antennae of Cynipoidea, which in most cases (39 of the studied species) bear just one SCo in each flagellomere; these sensilla have been also described as “pit organs”, in particular in Aculeata, because they are recessed into deep pits [9], [33], and as coeloconic sensilla type II [24]. In Cynipoidea, they were previously detected in *Aganaspis* spp. [49] (where they were named sensilla coeloconica type I), *T. rapae* [48] and *D. kuriphilus* [50]. In particular for *Aganaspis*, it was interesting that some flagellomeres can bear up to 6 SCo-A in cluster [49], a pattern that we found here only in Pediaspidini (Cynipidae). Here we provide the first evidence for their presence in most lineages of Cynipoidea, apparently lacking only in Liopteridae, Paraulacini, Qwaqwaiini, three *Synergus* and one figitid. The general morphology of SCo-A is similar among Cynipoidea, Braconidae [23], [24]–[26] and Chalcidoidea [73], [87], [88], while they have a slightly different morphology in Platygastroidea [69], [76], [85], [86], where they were sometimes described as “sensillum styloconicum” [55], [80].

The morphology of SCo-A in Cynipoidea is quite similar among species, and the main differences concern the number and position of SCo-A on a flagellomere. As for SP, also these characters related to SCo-A present very variable states within lineages. However, at least for one of the characters (23) we can attempt to propose an evolutionary trend. In particular, the relative size of SCo-A seems to have increase in Cynipidae (Fig. 1, Table 2). *Ibalia rufipes* Cresson possess very small SCo-A compared with flagellar width (as seen in the F_A_, apparently the only segment where it occurs in this species). All Figitidae have also small SCo-A (state 0); then, within Cynipidae, the basal clade Pediaspidini + Diplolepidini has a 0/1 state, and then SCo-A becomes larger in Eschatocerini (state 2) and presents a mix of states (0 to 2) in the remaining tribes (notably the state 2 only reappears in the more derived Cynipini).

Interestingly, because Pediaspidini have larger and more SCo-A than Diplolepidini, it seems that SCo-A, overall, cover more flagellar surface in species inducing galls in trees than in those inducing galls in herbs, perhaps in response to certain environmental/ecological pressure related with galling in woody substrates. On the other hand, the character related to the number of SCo-A per flagellomere alone appeared too variable within lineages to permit proposing any overall picture. Also interestingly, one observation (character 22) seems to contrast with the most recent phylogeny of Cynipidae (Fig. 1). In fact, herb-gallers from two distinct clades (Aylacini I + Aylacini II) had SCo-A far from the distal margin of the flagellomere, differently from their respective most closely-related lineages (Synergini I and Synergini II). We recognized also a smaller type of SCo (SCo-B), very similar to that observed in *Aganaspis* spp. [49] and *Cotesia* spp. (Braconidae) [24], [25]. Ultra-structural and electrophysiological investigations reveal that sensilla coeloconica in Hymenoptera have a thermo-hygroreceptive function [58], [90], [91]. We suggest a similar function in Cynipoidea.

The sensilla campaniformia (SCa) were described from both Aculeata (sometimes under the name of sensilla coelocapitula [33]) and different parasitic lineages [22], being similar in external morphology across the studied taxa. In the HAO portal, a campaniform sensillum is defined as an aporous sensillum without a hair like cuticular component [56]. Though this agrees in general with our definition, there are other types of sensilla that respond to such a definition, e.g. sensilla coelconica (see above). We suggest re-defining SCa more specifically as a domed, smooth, circular cuticular disk from the centre of which one (sometimes none, see below) small button-like knob emerges; such definition meets that for SCa found in many other hymenopteran groups (see below).

In Ichneumonoidea, SCa were observed in few Braconidae [74], though in most analysed species of this family it seems to be absent [18], [23], [25]–[27], [71], [75], [92]. Within Proctotrupomorpha excluding Cynipoidea, this sensilla type was observed in Scelioninae (Platygastroidea), Trichogrammatidae and Eupelmidae (Chalcidoidea) [22], [55], [88], [93], but is apparently absent in many other chalcidoid families such as Aphelinidae, Agaonidae, Pteromalidae, Eulophidae and Mymaridae [11], [29], [72], [76], [87]. In Cynipoidea, SCa were to date only observed in the figitid genus *Aganaspis* [49], so that here we add valuable information about its occurrence within this superfamily, and in particular we revealed that SCa may be much more widespread in this group (only 10 species apparently lack it in the studied sample, being half gall-inquilines), compared to the other Proctotrupomorpha and Ichneumonoidea. Sensilla campaniformia are also very widespread in the Aculeata [9], [31], [33]. Interestingly, within Hymenoptera, even when SCa is not present on the antennae, it can occur in other body parts, notably in the orbicula, i.e. a dorsal sclerite between the tarsal claws [94].

Sensilla campaniformia were considered in the past to be mechanoreceptors [31], [86]. However, Ochieng et al. [23] reported that SCa may serve a gustatory role due to the presence of a porous tip, while Dietz and Humphreys [30] exclude an olfactory function by amputation experiments, and reported that the porous central tip in the SCa of the honeybee (*Apis mellifera* L.) is involved in a gustatory function and is highly susceptible to humidity. Electrophysiological studies also suggest that SCa are thermo-hygroreceptors [91], [95]. In contrast to those hypotheses, Romani et al. [55] found SCa without the button-like knob observed in our study, as well as in many other observations on Hymenoptera (see above). These authors suspected that such sensilla could be involved in the release of the secretion of the antennal glands, and suggest that all the previously described SCa possessing a knob are actually SCo. Though we cannot exclude this possibility, here we consider our studied SCa homologous with the SCa described in many studies on a wide taxonomic range, due to their close similarity. This hypothesis should be tested in the future with histological observations.

Sensilla basiconica (SB), overall being cone-like, thick peg-structures setting into a shallow cuticular depression, are not uncommon in Hymenoptera, having being described for both Aculeata (where they are large and extremely abundant dorsally on the antennae) [9], [33], [60] and different parasitic lineages (where they are generally much smaller and less abundant in the antennae) (see below). The only definition associated with the term sensilla basiconica in the HAO portal refers to coeloconic sensillum of the galea [56], thus not to an antenna structure. We suggest including our definition, which is sufficiently wide to embark the known subtypes of SB found across Hymenoptera (see Discussion), within the antennal sensory system. In Cynipoidea, SB are also common, having being observed in 48 of the studied taxa (they lack in 5 species, including 3 figitids). The absence of SB in *Ganaspis* sp. here observed agrees with their absence in the other previously studied Eucoilinae [48], [49]. On the other hand, Romani et al. [50] did not report SB for *D. kuriphilus*, while we found such sensilla type in this cynipid. Sensilla basiconica present a wide range of sizes and shape in the non-Aculeata species studied so far, suggesting that this definition may include more than one type of sensilla. For example, in the genus *Encarsia* (Chalcidoidea: Aphelinidae), Viggiani and Mazzone [70] reported both “basiconic capitate sensilla” and “basiconic sensilla” (the latter seeming more similar to SB observed here for Cynipoidea). Even in about half of our studied species SB seem to occur in two sub-forms: apart from the typical form, they present a much smaller SB of about only 1.5 µm in length. In *Ooencyrtus phongi* Trjapitzin, Myartseva & Kostjukov (Chalcidoidea: Encyrtidae), SB have a peculiar morphology, being strongly bulbous in their distal part [71], which was not observed in any of the cynipoid species studied here. In a study on *Trichogramma australicum* Girault (Chalcidoidea: Trichogrammatidae), Amornsak et al. [22] found “basiconic capitate sensilla” resembling the SCo described for the eulophid *Sympiesis sericeicornis* Nees [96]. Within Proctotrupomorpha, this type of very bulbous SB has consistently been reported in many other Chalcidoidea [29], [72], [76], [87], [97], and closely resemble the “grooved peg sensilla” described in Platygastroidea [73], while, as already stated, it is absent in Cynipoidea (this study). Some “sensilla chaetica” described for Chalcidoidea, on the other hand, more closely resemble our described SB [11], [76]. Platygastroidea seem to have SB more similar to those found by us in Cynipoidea [81], [89]. In Ichneumonoidea, SB was found in some Braconidae, though with morphology very different from that we found in Cynipoidea, and different also from that found in other Hymenoptera, i.e. with a more trichoid, skinnier shape [23], [27], [74], [98]. Due to this elongate shape, some studies named these SB as “fluted basiconic sensilla” [18], while Bleeker et al. [24] and Barbarossa et al. [99] directly consider them as sensilla trichoidea. However, more typical SB also seem to occur in braconids, though they were probably classified with other names. For example, an examination of the study of Obonyo et al. [75] on *Cotesia* spp. reveals some types of sensilla, named “sensilla chaetica type 2 and 3” which closely resemble SB found in Cynipoidea. A similar case may concern the “sensillum trichodeum TP” described for *Cotesia* spp. by Bleeker et al. [24], which, due to its grooved surface, pores on the apex, and a socket, resemble the SB described by us for Cynipoidea.

The most probable function of SB is related with the olfaction, given its porous peg [100]–[102]; however, a hygro-, thermo- and mechanoreceptor-function was also suggested [103]. It is possible that SB involve a bi-modal function as chemo- and thermoreceptors [21], [47].

Sensilla trichoidea (ST) are by far the most diverse sensillar structures, including, at least in Cynipoidea, five morphologically distinct types, with three (ST-A, ST-B and ST-C) occurring in most or all species. Within Hymenoptera, including the other few Cynipoidea studied to date, it is very common to describe several types of ST in a single species, making comparisons across the literature difficult (see below). In the HAO portal, a trichoid sensillum (or seta or bristle) is a sensillum that is multicellular and consists of trichogen, tormogen, and sense cells and the cuticle secreted by and adjacent to the trichogen cell [56]. Such definition is too vague to indicate precisely a trichoid sensillum: first, there are more sensillar types which consist of multicellular structures (including trichogen cells), e.g. sensilla placoidea [51]; second, the HAO definition missed the most important external feature of a trichoid sensillum, i.e. its hair-like shape. We suggest defining sensilla trichoidea as any type of aporous, uniporous or multiporous sensilla with a hair-like structure. Subtypes could be then defined depending on pore numbers, length, and morphology of the hair and of hair insertion, but we did not attempt to suggest such a finer classification for the HAO portal. We also suggest that setae should not be considered as trichoid sensilla, but should indicate non-innervated hair-like structures without a sensing role, as proposed for Aculeata [31], [35].

Based on external morphology, we can report some cases of ST found in other parasitic species that clearly resemble the ST found in Cynipoidea. Sensilla trichoidea type A described here resemble the ST in *Ceratosolen solmsi marchali* Mayr (Chalcidoidea: Agaonidae) [72], the “S. trichodea TP” in *Aphidius rhopalosiphi* De Stefani-Perez (Ichneumonoidea: Braconidae) [26] and the “ST-UP” in *Encarsia guadeloupae* Viaggiani (Chalcidoidea: Aphelinidae) [76]. The peculiar ST-A found in triplet on the antennal apex of Eschatocerini resemble the mechanosensory hairs found on the antennal scape of the scilonid *Trissolcus basalis* (Wollaston) (Platygastroidea) [55]. Sensilla trichoidea type B of our study in Cynipoidea closely resemble the ST-I in *Aganaspis* spp. (Figitidae) [49] and the “long ST” of *T. rapae* (Figitidae) [48], where it also occurs in similar arrangement (i.e. laterally, in pairs at opposite positions on the distal part of the flagellomeres). Sensilla trichoidea type B also resemble the “ST_1_” of *Spathius agrili* Yang (Braconidae) [104], the “sensilla chaetica type 1” of *Cotesia* spp. [75], the “sensillum chaeticum” of *Telenomus reynoldsi* Gordh & Coker (Platygastroidea: Scelioninae) [79] and the uniporous gustatory sensillum of *T. basalis* (Scelioninae) [55]. Sensilla trichoidea type C were observed in our study in all taxa, and represent the more numerous sensillar type on the cynipoid antennae, as also reported for other non-Aculeata lineages [55]. These sensilla resemble the “SCh-7” of *O. phongi* (Chalcidoidea: Encyrtidae) [71] and the aporous mechanosensory hairs reported for different parasitoid lineages [55]. Sensilla trichoidea type D, which were rare in Cynipoidea, having being detected in only 12 taxa, resemble the “ChS-1” of *C. solmsi marchali* [72]. Sensilla trichoidea type E were found to be extremely rare in Cynipoidea (5 taxa in this study); we were unable to find close similarity between its peculiar “twisted-furrowed” shape and any ST described for other Hymenoptera. No apparent effect of phylogeny on the occurrence of the different types of ST can be observed, and no evident association of their presence with certain life-history traits appeared.

Sensilla trichoidea, as a general category including the five types of hair-like structures here described, and in particular ST-C, cover the antennae of Cynipoidea with density varying from low to high, without an apparent effect of phylogeny on such variation. For example, *Ganaspis* sp. and *Acanthaegilips* sp., both in the Figitidae, had, respectively, a very low and a very high ST density along flagellomeres. However, at least it seems that basal Cynipoidea (Liopteridae and Ibaliidae) tend to have higher ST density; then, both within Figitidae and within Cynipidae, ST density seemed to have increased and decreased several times; in particular within Cynipidae, the group composed by Aylacini I+II and Synergini I+II seeming to have higher ST density than Cynipini and the other tribes of wood-gallers (Table 2).

The functions of ST are difficult to hypothesize without a detailed study on their internal structure. In Cynipoidea, it is suggested that the long ST (as the ST-B in our study) are chemo-receptors by contact (gustatory) and the shorter ST (like our ST-A and ST-C) are mechanoreceptors [48], [50], [55].

The large volcano sensilla (SLV) and the large disc sensilla (SLD) described here for Paraulacini and Plectocynipinae, respectively, are peculiar structures which we were unable to find in any other Hymenoptera studied to date. We suggest introducing these terms and definitions in the HAO portal, expressly referring to Paraulacini and Plectocynipinae.

Because SLD were observed in two different genera of Plectocynipinae, this may represent a synapomorphy for this subfamily. On the other hand, for Paraulacini, we only could analyse one species of *Cecinothophagus*, and preliminary unpublished observations (J. L. Nieves-Aldrey) on other species of this genus suggest that all possess a SLV. For the only other genus of Paraulacini, *Paraulax*, available data are insufficient to clearly assess if a SLV occurs on the antennal clava [65]. Thus, it is unclear at the moment if SLV may represent a synapomorphy for *Cecinothophagus* or for Paraulacini. We cannot attempt at the moment to hypothesize the function of SLD and SLV. Interestingly, these apical structures were only found in gall-inquiline species (and in one species with uncertain biology), doubtfully assigned to inquilines or parasitoids, of galls induced by chalcids on *Nothofagus* trees (Nothofagaceae). These species belong to two morphologically aberrant phylogenetic lineages, the Paraulacini and the Plectocynipinae, which are endemic of the temperate Neotropical region (Chile and Argentina) [65], [105], though it is not possible at the moment claim for a link between their presence and gall-attacking strategy.

In conclusion, we found a great variability in antennal morphology and even more in the antennal sensillar equipment within Cynipoidea. Such variability make it difficult to propose the general use of sensillar characters in taxonomic studies, though at least some characters may help to distinguish some cynipid tribes with special features on the antennae. Some evolutionary trends for certain sensilla types can be preliminary suggested, but what is required is a larger sample size, in particular for Figitidae. On the other hand, the observed variability may perhaps have some links with the different life-history traits (in particular SCo size with host plant), but overall the sensillar equipment of Cynipoidea is a complex result of different interacting pressures and evolutionary histories, which need further investigation to be clarified.

## Supporting Information

Figure S1 SEM pictures showing examples of character states for characters related with antennal morphology (1–5).
(TIF)

Figure S2 SEM pictures showing examples of character states for characters related with sensilla placoidea (11–18).
(TIF)

Figure S3 SEM pictures showing examples of character states for characters related with sensilla coeloconica type A (19, 21–23).
(TIF)

Figure S4 SEM pictures showing examples of character states for characters related with length and density of sensilla trichoidea (33–34).
(TIF)
