# Supplementary figures and images for: Diverse Filters to Sense: Great Variability of Antennal Morphology and Sensillar Equipment in Gall-Wasps (Hymenoptera: Cynipidae)
Source: PLoS One. 2014 Jul 8;9(7):e101843. doi: 10.1371/journal.pone.0101843 (PMC4087010; doi:10.1371/journal.pone.0101843)

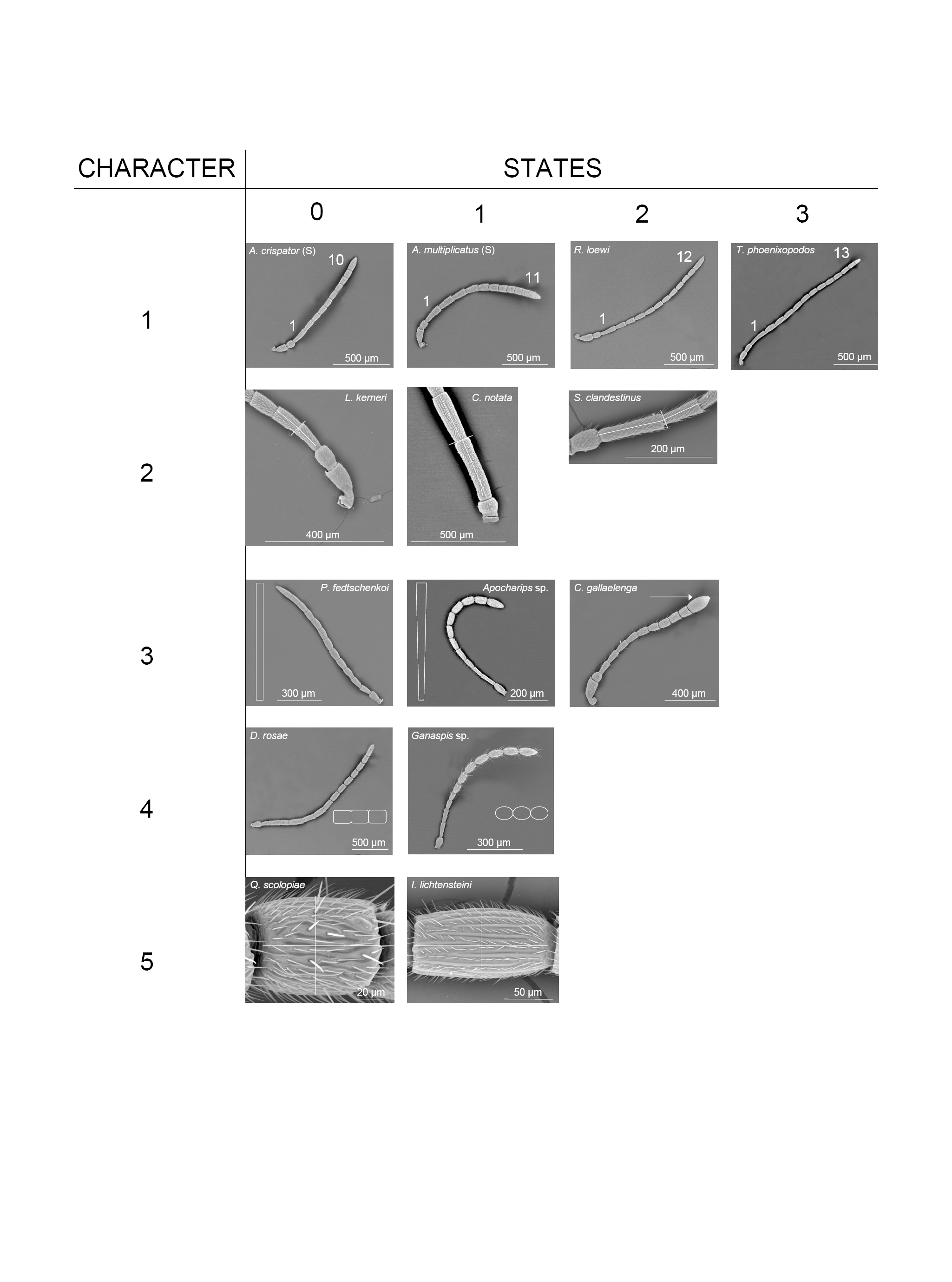

Supplement: Figure S1 — SEM pictures showing examples of character states for characters related with antennal morphology (1–5). (TIF) [file pone.0101843.s001.tif]

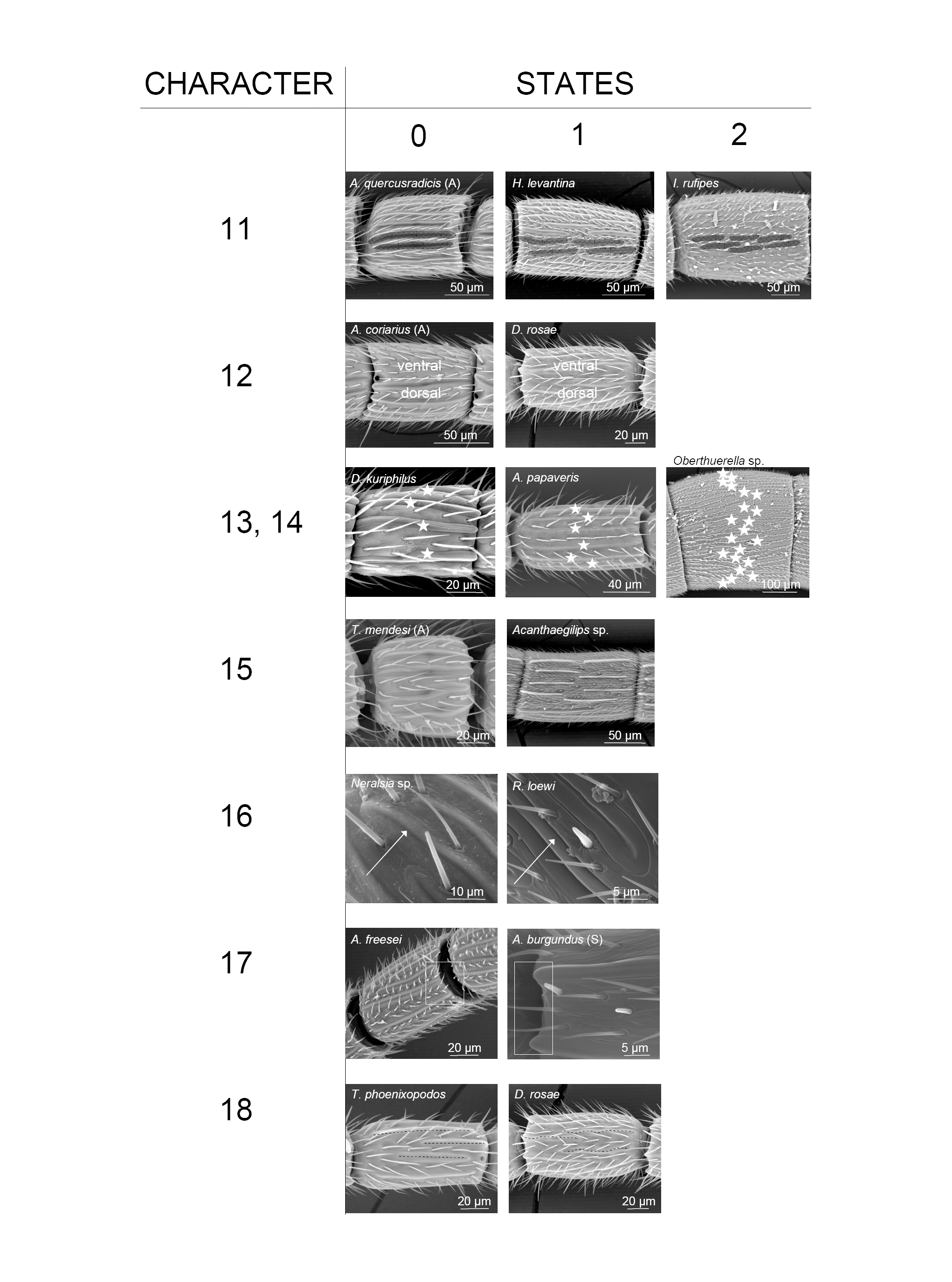

Supplement: Figure S2 — SEM pictures showing examples of character states for characters related with sensilla placoidea (11–18). (TIF) [file pone.0101843.s002.tif]

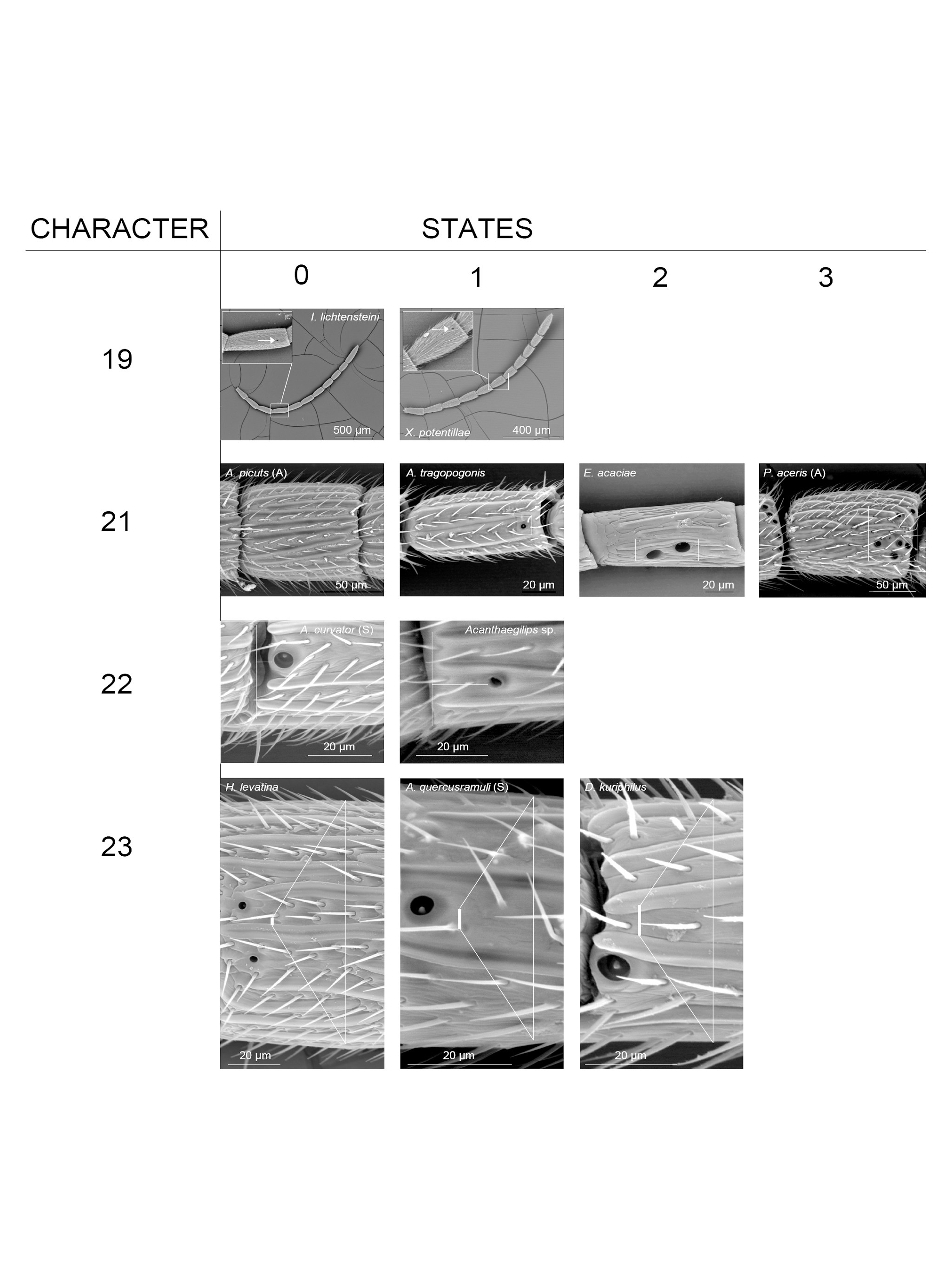

Supplement: Figure S3 — SEM pictures showing examples of character states for characters related with sensilla coeloconica type A (19, 21–23). (TIF) [file pone.0101843.s003.tif]

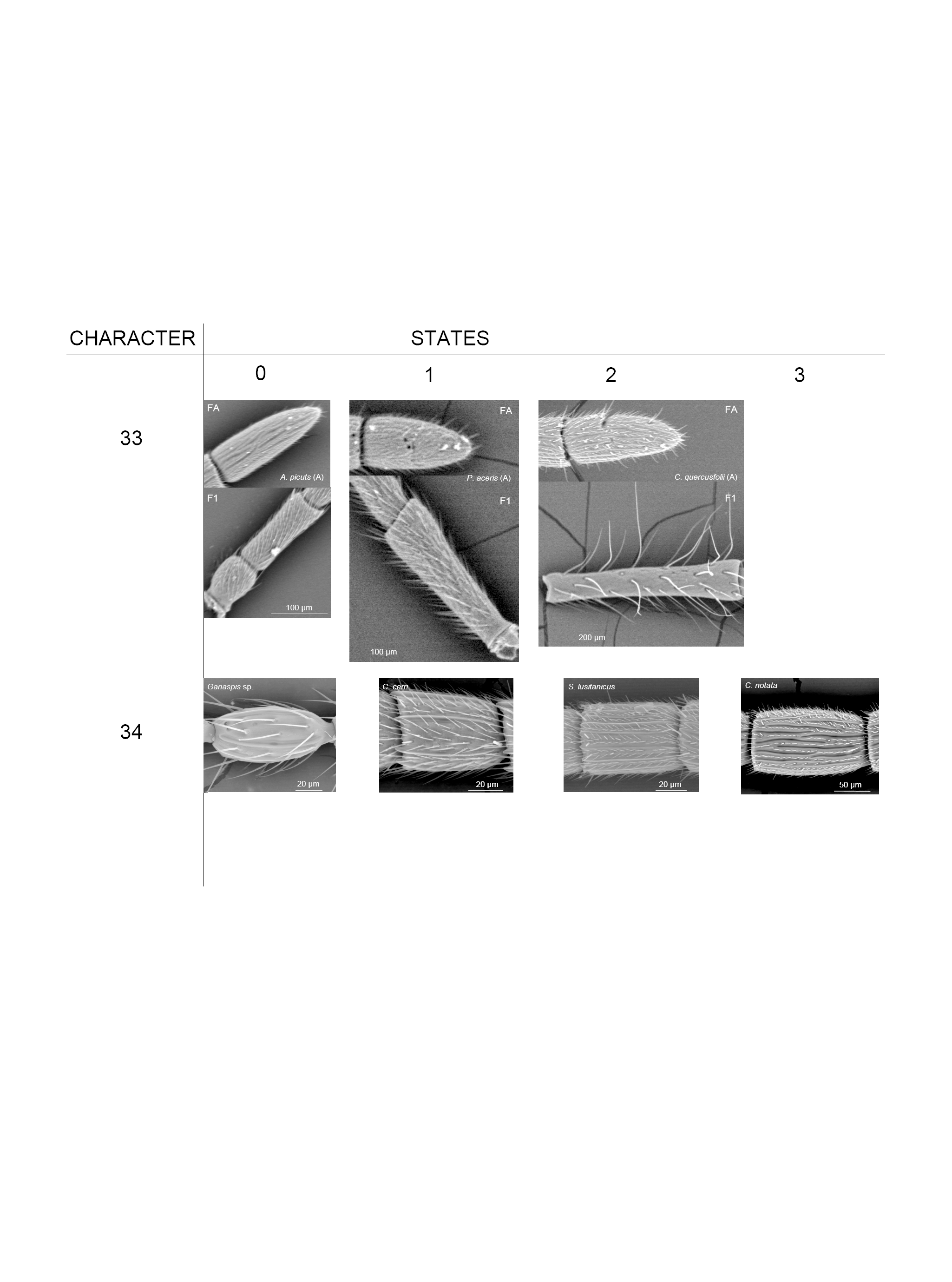

Supplement: Figure S4 — SEM pictures showing examples of character states for characters related with length and density of sensilla trichoidea (33–34). (TIF) [file pone.0101843.s004.tif]
